# On the edge of Bantu expansions: mtDNA, Y chromosome and lactase persistence genetic variation in southwestern Angola

**DOI:** 10.1186/1471-2148-9-80

**Published:** 2009-04-21

**Authors:** Margarida Coelho, Fernando Sequeira, Donata Luiselli, Sandra Beleza, Jorge Rocha

**Affiliations:** 1 IPATIMUP, Instituto de Patologia e Imunologia Molecular da Universidade do Porto, R Dr Roberto Frias s/n, 4200-465 Porto, Portugal; 2 Departamento de Zoologia e Antropologia, Faculdade de Ciências da Universidade do Porto, Praça Gomes Teixeira, 4099-002 Porto, Portugal; 3 CIBIO, Centro de Investigação em Biodiversidade e Recursos Genéticos, Campus Agrário de Vairão, 4485-661 Vairão, Portugal; 4 Dipartimento di Biologia Evoluzionistica Sperimentale, Universita di Bologna, Via Selmi, 3 Bologna, Italy

## Abstract

**Background:**

Current information about the expansion of Bantu-speaking peoples is hampered by the scarcity of genetic data from well identified populations from southern Africa. Here, we fill an important gap in the analysis of the western edge of the Bantu migrations by studying for the first time the patterns of Y-chromosome, mtDNA and lactase persistence genetic variation in four representative groups living around the Namib Desert in southwestern Angola (Ovimbundu, Ganguela, Nyaneka-Nkumbi and Kuvale). We assessed the differentiation between these populations and their levels of admixture with Khoe-San groups, and examined their relationship with other sub-Saharan populations. We further combined our dataset with previously published data on Y-chromosome and mtDNA variation to explore a general isolation with migration model and infer the demographic parameters underlying current genetic diversity in Bantu populations.

**Results:**

Correspondence analysis, lineage sharing patterns and admixture estimates indicate that the gene pool from southwestern Angola is predominantly derived from West-Central Africa. The pastoralist Herero-speaking Kuvale people were additionally characterized by relatively high frequencies of Y-chromosome (12%) and mtDNA (22%) Khoe-San lineages, as well as by the presence of the -14010C lactase persistence mutation (6%), which likely originated in non-Bantu pastoralists from East Africa. Inferred demographic parameters show that both male and female populations underwent significant size growth after the split between the western and eastern branches of Bantu expansions occurring 4000 years ago. However, males had lower population sizes and migration rates than females throughout the Bantu dispersals.

**Conclusion:**

Genetic variation in southwestern Angola essentially results from the encounter of an offshoot of West-Central Africa with autochthonous Khoisan-speaking peoples from the south. Interactions between the Bantus and the Khoe-San likely involved cattle herders from the two groups sharing common aspects of their social organization. The presence of the -14010C mutation in southwestern Angola provides a link between the East and Southwest African pastoral scenes that might have been established indirectly, through migrations of Khoe herders across southern Africa. Differences in patterns of mtDNA and Y-chromosome intrapopulation diversity and interpopulation differentiation may be explained by contrasting demographic histories underlying the current female and male genetic variation.

## Background

Among the complex series of demographic events that shaped the patterns of human genetic variation in Africa, the massive dispersal of Bantu-speakers stands as one of the most impressive examples of human migration. Both linguistic and archeological evidences suggest that the spread of Bantu languages started about 4000 years ago in the adjacent grasslands of Cameroon-Nigeria and involved large movements of farmers carrying an agricultural tradition especially well-suited to the climate conditions prevailing in subequatorial Africa [1,2]. According to a widely accepted dispersion model, one major population movement involved the expansion of ancestors of East Bantu speakers along the northern fringe of the African rain forest into the interlacustrine areas surrounding Uganda [1-3]. Another important movement is thought to be linked to the early penetration of ancestors of West Bantu speakers into the wet coastal areas of the central African forest, beyond the Cameroon plateau [2]. More recent major expansions would include the migrations of West and East Bantu speakers into the dry territories located beyond the southern borders of the rain forests, which eventually culminated with the diffusion of Bantu languages across southern Africa [1,2]. However, this basic representation of the major trends of Bantu dispersals has not remained unchallenged [3-5], and many specific details of the migration dynamics leading to the emergence of widespread Bantu-speaking communities are still poorly understood [3].

Genetic data have great potential for unraveling the complex population history underlying Bantu expansions, but there are a number of difficulties related to sampling coverage and parameter estimation that need to be overcome. So far, most of the available genetic information has been gathered in phylogeographic studies of Y-chromosome and mitochondrial DNA (mtDNA) variation. These studies identified several mtDNA haplogroups likely to be associated with the Bantu migrations that trace their ancestries to different geographic regions of Africa [6]. In contrast, the great majority of Bantu Y-chromosome lineages were found to belong to a single widespread haplogroup (E3a), which seems to have overrun most pre-existing diversity [7-9]. Recently, a few studies have begun to address more detailed aspects of regional mtDNA variation by increasing both the resolution of sequence data and the density of population sampling [10,11]. However, in spite of this progress, current understanding of Bantu expansions is still hampered by lack of sampling of crucial regions in subequatorial Africa. The area of Angola, in particular, has remained persistently underrepresented in most studies of African genetic variation [12]. Although new genetic data has become available from Kimbundu and Bakongo speakers from northern Angola and Cabinda [13,14], no information exists on the broad area encompassing the dry woodlands to the south of the Cuanza river, which is critical for understanding the push of West Savanna Bantu-speaking peoples out of the rain forest into the arid steppes of southwestern Africa.

Being exposed to the effects of the Benguela current, southern Angola provided a new environment characterized by increasing levels of aridity that challenged the progression of the agricultural lifestyle that had predominated in the well irrigated lands of the Congo basin [1,2]. Faced with this environmental shift, some groups, like the Ovimbundu, settled the high grounds of the Bié plateau where they could find areas of relatively fertile soil and higher rainfall [2]. In the coastal areas, and further to the south, settlements had to be limited to major river valleys and subsistence economies became increasingly dependent on cattle raising. The Herero, the Ovambo and the Nyaneka-Nkhumbi are examples of such Bantu groups in southwestern Angola and form a broad cultural and economic cluster relying on cattle raising to various degrees [15,16]. Among them, the Herero are the most exclusively pastoral of all Bantu peoples from southwestern Africa and penetrated well into the arid regions of the Namib Desert where they shared their mode of life with neighboring non-Bantu Khoe cattle herders [17]. This cultural and geographical proximity between Bantu and Khoisan-speaking groups poses intriguing questions about the development of the Southwest African pastoral scene and the nature of the interactions between the vanguard of West Bantu speakers and the non-Bantu peoples from the desert. For example, the role played by Khoe herders in the adoption of the present pastoral specialization of Bantu speakers is still not clear [18]. Moreover, the relative isolation of Southwest Africa from the major East African pastoral centers represents an important challenge for the identification of the migration routes that led to the emergence of a cattle-herding zone in the southwestern periphery.

Here we present an analysis of the western edge of the Bantu expansions based in the study of Y-chromosome, mtDNA and lactase persistence genetic variation in West Savanna Bantu-speaking groups from southwestern Angola. We analyzed the data in the context of regional and continental genetic diversity by assessing the differentiation between these populations and their levels of admixture with Khoisan-speaking groups, and by examining the relationship between southwestern Angola and other areas of Africa. Furthermore, we combined our dataset with published data on Y-chromosome and mtDNA variation from Southeast Africa to explore a general isolation with migration (IM) model [19,20] and infer key demographic parameters underlying the history of Bantu expansions.

## Methods

### Population Samples

**Figure 1:**
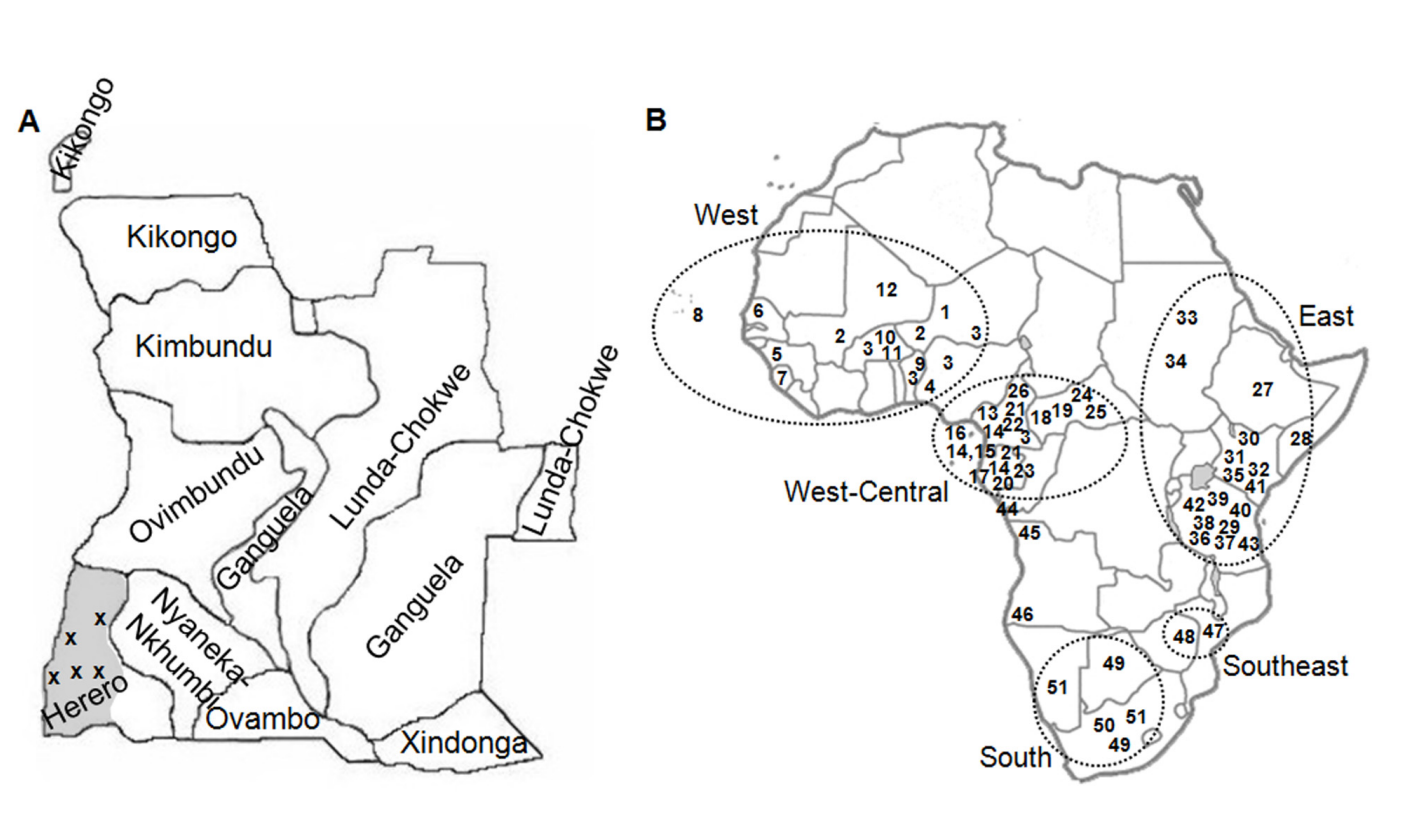
**Major ethnolingusitic groups from Angola and population samples used in this study**. A) Map of Angola depicting the core areas of the country's major ethnolinguistic groups and sampled locations in the Namibe province (modified from [21]). The area encompassing the sampled locations is shaded. B) Map of Africa with the approximate locations of the population groups used in the present analysis. Populations are coded with numbers. The correspondence between numbers and populations is given in Additional files 4 and 5. Major geographic regions are encircled.

Buccal swabs were collected from 54 Kuvale, 153 Nyaneka-Nkhumbi, 21 Guanguela, 96 Ovimbundu, and 45 Bantu-speaking individuals with other ethnic affiliations. Individuals were grouped according to self-identified ethnicity, and only samples from unrelated individuals were included in the study. Samples were collected after informed consent in donors' hometowns and villages located in the administrative province of Namibe, southwestern Angola (Figure 1A). Besides including the original core area of Herero-speaking peoples, the province is presently inhabited by different ethnic groups due to relatively recent migrations from surrounding areas.

The groups included in our sample represent West-Savanna Bantu-speaking populations [22] from the southwestern edge of the Bantu expansions (Figure 1A) and rely on different combinations of agricultural and pastoral lifeways. The Ovimbundu form the largest ethnolinguistic cluster in Angola, making up 35% of the total population. Their original core area was located in the Bié plateau, but they underwent a series of southward expansions that considerably enlarged their territory and are presently one of the major groups inhabiting Namibe [15,16] (Figure 1A). Traditionally, most Ovimbundu groups practiced mixed farming and kept livestock. However, cattle raising was not crucial for subsistence and few families owned large herds [15]. The Nyaneka-Nkhumbi-speaking groups, who have also spread to present-day Namibe, originally settled the area located West from the middle Cunene River, including the Huíla plateau in the eastern limit of the Namibe province (Figure 1A). These peoples are agro-pastoralists that depend in part on cultivation, but keep large cattle herds and use dairying products as an important source of subsistence [16,23]. The Kuvale people dwell in the arid lowland areas of the Namibe province and are one of the most representative Herero-speaking groups from Angola [17] (Figure 1A). Like other groups included in the Herero cultural division, they are semi-nomadic cattle herders and rank among the most exclusively pastoral peoples of southwestern Africa. The Ganguela-speaking peoples originally settled southeastern Angola, which is well removed from Namibe (Figure 1A). However, during the Angolan civil war many Ganguela families flew to neighboring countries and to other regions of Angola, including Namibe. The Ganguela originally included a number of scattered farming communities that were split by the southern expansion of the Chokwe peoples in the 19^th ^century. The populations that remained in the western side of the Chokwe penetration progressively adopted cattle and became mixed farmers [16].

### Laboratory methods

#### mtDNA

We have sequenced both hypervariable segments I (HVS-I; positions 16024–16400) and II (HVS-II; positions 73–340) of the mtDNA control region. MtDNA sequencing was performed as described previously [14]. All HVS-I and HVS-II sequences are shown in Additional file 1. To assign mtDNA sequences to previously defined haplogroups, we initially followed established criteria based on HVS-I sequence variation [6,24] updated as recently discussed [25]. Occasional ambiguities in these assignments were resolved by additional typing of a selected set of four diagnostic restriction fragment length polymorphisms (RFLPs): 3592 *Hpa*I (absent in L3), 2349 *Mbo*I (present in L3e), 10084 *Taq*I (present in L3b) and 8616 *Mbo*I (absent in L3d). After this initial assignment step, we used the available information on HVS-I and HVS-II variation provided by published complete mtDNA sequences to refine and/or rename the classifications according to the most recently updated mtDNA phylogeny [26] (see Additional file 1). For the sake of comparison we refer to the HVS-I-based nomenclature throughout the article.

#### Y-chromosome

To characterize the nonrecombining portion of the Y-chromosome (NRY) we genotyped 9 unique event polymorphisms (UEPs; M2, M35, M60, M91, M112, M150, M213, YAP, SRY4064) and 11 short tandem repeats (STRs; DYS19, DYS389I, DYS389II, DYS385, DYS390, DYS391, DYS392, DYS393, DYS437, DYS438, and DYS439). The DYS385 locus consists of a duplicated tetranucleotide STR region and was omitted from some analyses. Except for YAP, UEPs were typed by direct sequencing of PCR products. Primer sequences and protocols are provided upon request. Short tandem repeats were typed with the Promega Powerplex Y System. All Y-chromosome combined haplotypes, defined by UEP and STRs, are shown in Additional file 2. For the sake of comparison NRY haplogroups based on the UEP variation were named according to the Y-chromosome Consortium guidelines [27,28], but we also provide haplogroup names according to a most recent update [29], which in our dataset essentially involves the renaming of haplogroup E3a as E1b1a (see Additional file 2).

### Lactase persistence

Lactase persistence was screened by direct sequencing of a 359 bp PCR fragment located within intron 13 of the MCM6 gene, which contains all single nucleotide polymorphisms (SNPs) that have been so far associated with lactase persistence in human populations: G/C -14010; T/G -13915; C/T -13910; and C/G -13907 [30-32] (see Additional file 3 for typing details). In addition to the southwestern Angolan sample, we further typed the lactase persistence-associated SNPs in a total of 111 Bantu speaking individuals belonging to 11 different population groups from Mozambique: 3 Chopi, 4 Chwabo, 19 Makhwa, 15 Makonde, 15 Ndau, 11 Nyanja, 15 Ronga, 2 Sena, 15 Shangaan, 1 Shona and 11 Tswa.

### Data analyses

Summary statistics for mtDNA and Y-chromosome haplotype variation, and Tajima's *D *and Fu's *Fs *tests were calculated and performed with the ARLEQUIN 3.11 software package [33]. Analyses of molecular variance (AMOVA) to evaluate the apportionment of genetic variation were also performed using ARLEQUIN 3.11. Correspondence analysis based on mtDNA and Y-chromosome haplogroup frequencies was performed using the POPSTR program [34].

Population cross-comparisons for the mtDNA data were restricted to the 16090–16365 HVS-I sequence range and were based in an assembled dataset comprising approximately 5400 mtDNA profiles from 73 populations (Figure 1B and Additional file 4). For the NRY data, cross-population comparisons were based either on UEP-defined haplogroups or on higher resolution haplotypes defined by a subset of 7 STR loci (DYS19, DYS389I, DYS389II, DYS390, DYS391, DYS392, DYS393) common to all samples assembled in a database of about 5072 haplotypes from 72 populations (see Additional file 5). Networks of NRY haplotypes and mtDNA sequences were constructed using the NETWORK 4.5 software [35]. For NRY haplotypes, the reduced-median [36] and median-joining [37] algorithms were applied sequentially and differential microsatellite weighting was used to resolve extensive reticulation at microsatellite loci. Weights for each microsatellite were inversely proportional to the ratio of the variance displayed by each marker within the respective haplogroups and the average variance value across loci in those haplogroups. For mtDNA sequences, the median-joining algorithm [37] was used without further weighting. Ages of mtDNA and NRY lineages were estimated with the ρ (rho) statistic [38] using NETWORK 4.5, assuming 25 years per generation, a mtDNA control region mutation rate of μ = 7.55 x 10^-6 ^per nucleotide per generation, based in a recent Bayesian estimate [39], and the following NRY-STR per generation mutation rates [40]: μ_DYS19 _= 0.0017; μ_DYS389I _= 0.0019; μ_DYS389II _= 0.0023; μ_DYS390 _= 0.0023; μ_DYS391 _= 0.0035; μ_DYS392 _= 0.0006; μ_DYS393 _= 0.0007.

Admixture proportions were estimated with the ADMIX2.0 program [41]. MtDNA-based estimates were calculated from haplogroup frequencies without taking into account molecular distances between haplogroups. NRY-based estimates were calculated from the frequency of haplotypes defined by STR loci DYS19, DYS389I, DYS389II, DYS390, DYS391, DYS392, and DYS393, not taking into account molecular distances between haplotypes.

We have also attempted to infer the key demographic parameters of Bantu expansions by analyzing our NRY and mtDNA data from southern Angola together with additional data from Southeast Africa (see Additional files 4 and 5) within the framework of a general isolation with migration (IM) model [19,20], using the IMa program [42]. The IM model describes the historical demographic properties of two related populations that may have varied in size after diverging from a single ancestral population, with bidirectional migration occurring at constant rate following the initial split [19,20]. Thus, we reasoned that this framework could be applied to populations located in the two opposite edges of the Bantu expansions in order to analyze the split between the eastern and western streams of Bantu dispersals after a common origin in an area likely to be located in West-Central Africa (see Additional file 6). The model has six parameters whose posterior probability distributions can be estimated by using the Markov chain Monte Carlo (MCMC) approach implemented in the IMa computer program [42]: effective population sizes for both extant (N_1 _and N_2_) and ancestral (N_A_) populations, time since divergence (t) and migration rates in both directions (m_1 _and m_2_). These demographic terms are obtained by conversion from estimated basic parameters that are scaled by the mutation rate (per locus per generation): θ_A _= 4N_A _μ; θ_1 _= 4N_1 _μ; θ_2 _= 4N_2 _μ; *t *= tμ; *m*_1 _= m1/μ; *m*_2 _= m2/μ. The mtDNA dataset consisted of 724 HVS-I 276 bp-long sequences ranging from positions 16090 to 16365, including 358 sequences from southwestern Angola, collected in the present study, and 366 assorted sequences from different ethnic groups from Mozambique and Zimbabwe (see Additional file 4). The NRY data consisted of 348 haplotypes defined by 7 STR loci totaling 236 Y chromosomes from the present Angolan sample and 112 chromosomes from Mozambique (see Additional file 5). Parameter conversions were done by using the aforementioned mtDNA control region and NRY STR mutation rates. After preliminary runs to determine plausible uniform prior ranges, the IMa program was run for at least 10 million steps after 100000 steps of burn-in with 8 Metropolis-coupled chains, with geometric heating. For each dataset at least two independent replicates were performed using the same running options and a different random seed to assess convergence of the parameter estimates. MtDNA sequences were assumed to mutate under the Hasegawa-Kishino-Yano (HKY) finite sites mutation model [43]. Mutation in NRY STRs was modeled by the stepwise mutation model (SMM). The mode of each marginal posterior distribution generated by the program was considered a point estimate of the corresponding parameter value. Reported parameter estimates are means from replicate runs.

## Results

### mtDNA

#### Haplotype and sequence diversity

Summary statistics for mtDNA HVS-I sequence diversity are presented in Table 1. The estimated levels of sequence variation in the whole sample from Namibe (θ_k _= 85; π = 0.025; H = 0.986) are within the range found in other Sub-Saharan regions [24]. However, diversity indices suggest that subsistence economy and population history had a measurable impact in the demography of the peoples from southern Angola. The semi-nomadic Herero-speaking Kuvale pastoralists display the lowest value of θ_k _(= 14.61), and fail to reveal any significant signal of population expansion, as reflected by the nonsignificance of both Tajima's D and Fu's Fs tests (Table 1). By contrast, the Ovimbundu and the Nyaneka-Nkhumbi agro-pastoralist groups exhibit higher levels of haplotype diversity and have significantly negative Fs values that are consistent with a history of demographic expansion. In order to confirm that the differences in Fs values were not influenced by sample size, we recalculated the Fs values in random subsamples of the Nyaneka-Nkhumbi and the Ovimbundu that matched the lower sample sizes of the Ganguela (N = 21) and the Kuvale (N = 54). For N = 21, Fs values remained significant for the Ovimbundu (Fs = -6.57; P = 0.01) but became nonsignificant for the Nyaneka-Nkhumbi (Fs = -3.97; P = 0.06). With N = 54, Fs values remained significant both for the Ovimbundu (Fs = -24.42; P = 0.00) and for Nyaneka-Nkhumbi (Fs = -11.40; P = 0.008), suggesting that the absence of traces of population expansion in the Kuvale is not attributable their lower sample size.

**Table 1:** MtDNA HVS-I sequence diversity in populations from southwestern Angola

Population	N	k	H	θk	θs	π	Tajima's D	Fu's Fs
		(k/N)	(SD)	(95% CI)	(SD)	(SD)	(P)	(P)
Kuvale	54	23	0.937	14.61	11.19	0.026	-0.45	-2.04
		(0.42)	(0.017)	(8.43–25.05)	(3.37)	(0.013)	(0.37)	(0.29)
Ganguela	21	16	0.962	28.92	10.28	0.025	-0.37	-3.76
		(0.76)	(0.030)	(12.08–73.02)	(3.76)	(0.013)	(0.38)	(0.06)
Nyaneka-Nkhumbi	153	73	0.982	54.11	12.67	0.024	-0.93	-24.44
		(0.48)	(0.003)	(38.93–75.04)	(3.18)	(0.012)	(0.16)	(0.00)
Ovimbundu	92	61	0.987	77.83	13.74	0.024	-1.10	-24.61
		(0.66)	(0.004)	(50.90–120.09)	(3.70)	(0.012)	(0.13)	(0.00)

Total	365	142	0.986	84.92	15.29	0.025	-1.12	-24.01
		(0.39)	(0.002)	(67.85–106.00)	(3.33)	(0.013)	(0.10)	(0.00)

N, number of sequences; k, number of different haplotypes; H, haplotype diversity; θ, mutation drift statistic calculated from the number of different haplotypes (θk) and number of segregating sites (θs); π, nucleotide diversity. The total sample includes 45 additional sequences from other groups.

#### Haplogroup composition

To investigate the relationship between Namibe and other African populations, a correspondence analysis based on mtDNA haplogroup frequencies was performed using contextual samples from different Sub-Saharan regions (Figure 1B; Additional file 4). The southern African Khoe-San samples distort the correspondence analysis (data not shown) due to their genetic uniqueness (high frequencies of haplogroups L0d/k) and were excluded from further analysis in order to achieve better resolution of the genetic relationships among populations.

**Figure 2:**
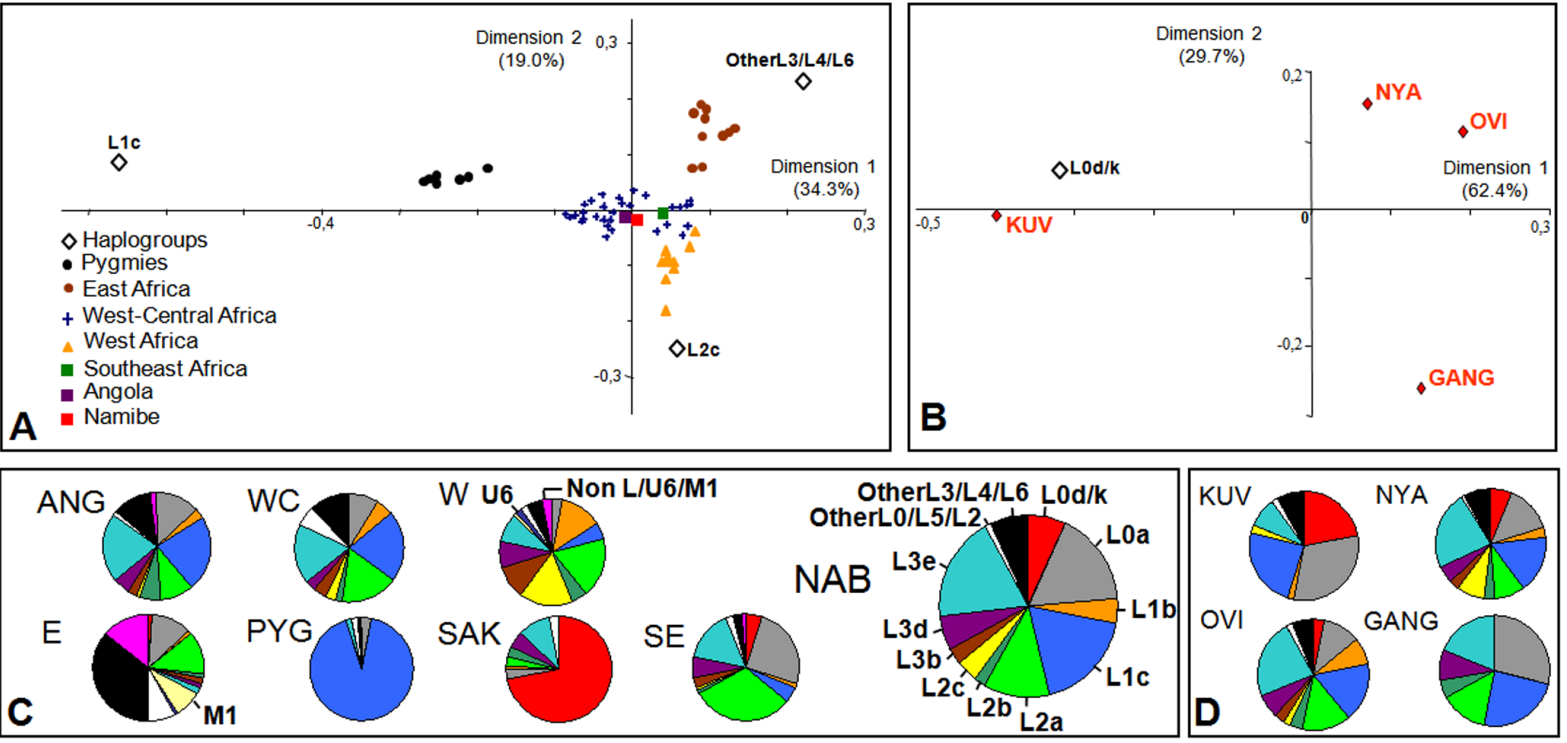
**MtDNA haplogroup variation in southwestern Angola and other African populations**. A) and B) Correspondence analysis plots based on haplogroup frequency profiles from several African populations (A) and different ethnolinguistic groups from Namibe (B). Percentages in parentheses indicate the total fraction of the genetic variation that was captured by each dimension. Geographic regions were defined as in Figure 1B. Populations from Mozambique and Zimbabwe were pooled into a single Southeast Africa group. Angola includes samples from Cabinda and Luanda. Namibe includes all groups sampled in this study. C) MtDNA haplogroup frequencies in the Namibe province and in other African population groups. D) MtDNA haplogroup frequencies in the four population groups sampled in the Namibe province. Haplogroup frequencies were broken down as in [25]. ANG = Angola (Luanda + Cabinda); WC = West-Central Africa; W = West Africa; E = East Africa; PYG = Pygmies; SAK = South Africa Khoe-San; SE = Southeast Africa; NAB = Namibe; KUV = Kuvale; OVI = Ovimbundu; NYA = Nyaneka-Nkhumbi; GANG = Ganguela.

Figure 2A shows the results of the correspondence analysis after exclusion of the Khoe-San samples. To facilitate graphical display, populations from Southeast Africa, which are known to be genetically homogeneous [6], were pooled into a single group. Samples from the northern Angolan regions of Cabinda and Luanda (here designated as Angola) were also combined. The pooled sample from Namibe lies in a cluster including most West-Central African populations, Angola and Southeast Africa. Pygmies display unusually high frequencies of haplogoup L1c and are clear genetic outliers. Despite some genetic continuity between West-Central and West Africa, the geographic regions of West-Central, West and East Africa seem to be well correlated with mtDNA variation, as populations from different regions tend to cluster around different coordinates. Furthermore, when populations were classified by four major geographic regions (West, East, Southeast and West-Central Africa) and two additional outlier ethnic groups (Pygmies and southern African Khoisan-speakers), AMOVA analysis showed that 14.2% of the variability lies between groups, 4.8% among populations within groups and 81% within populations (all P values <0.01).

Most mtDNA haplotypes that are commonly found in Sub-Saharan populations were also observed in Namibe (see Additional file 1; Figure 2C). The most frequent (>5%) haplogroups were L0d (6%), L0a1 (9%), L0a2 (8%), L1c1 (8%), L1c2 (7%), L2a1 (10%), L3e1 (9%), L3e2 (7%) and L3f (6%). Haplogroup L1c1a, which is typical of Pygmy populations from Central Africa [10,11], was virtually absent from our sample (see Additional files 1; Figure 2C). The relatively high frequency of the typical Khoe-San L0d haplogroup contrasts with previous findings from northern Angola [13,14] but compares with observations in Bantu groups from Southeast Africa [6,44]. However, this haplogroup is not evenly distributed in the Namibe samples and reaches much higher frequencies in the Kuvale (22%; Figure 2D) than in other groups. When correspondence analysis is focused on the four populations from Namibe (Figure 2B) the genetic peculiarity of the Kuvale caused by the high frequency of L0d becomes obvious.

### Patterns of lineage sharing

In order to analyze the likely origin of mtDNA sequences from southwestern Angola, we used the comparative mtDNA African dataset (see Figure 1B and Additional file 4) to study the patterns of HVS-I lineage sharing between Namibe and other Sub-Saharan populations. Although restriction of population cross-comparisons to the HVS-I control region increases the number of available samples, it is important to note that some matches may involve sequences that are phylogenetically unrelated. However, these cases are expected to seriously bias the conclusions only if convergence episodes are non-randomly distributed across lineages.

**Figure 3:**
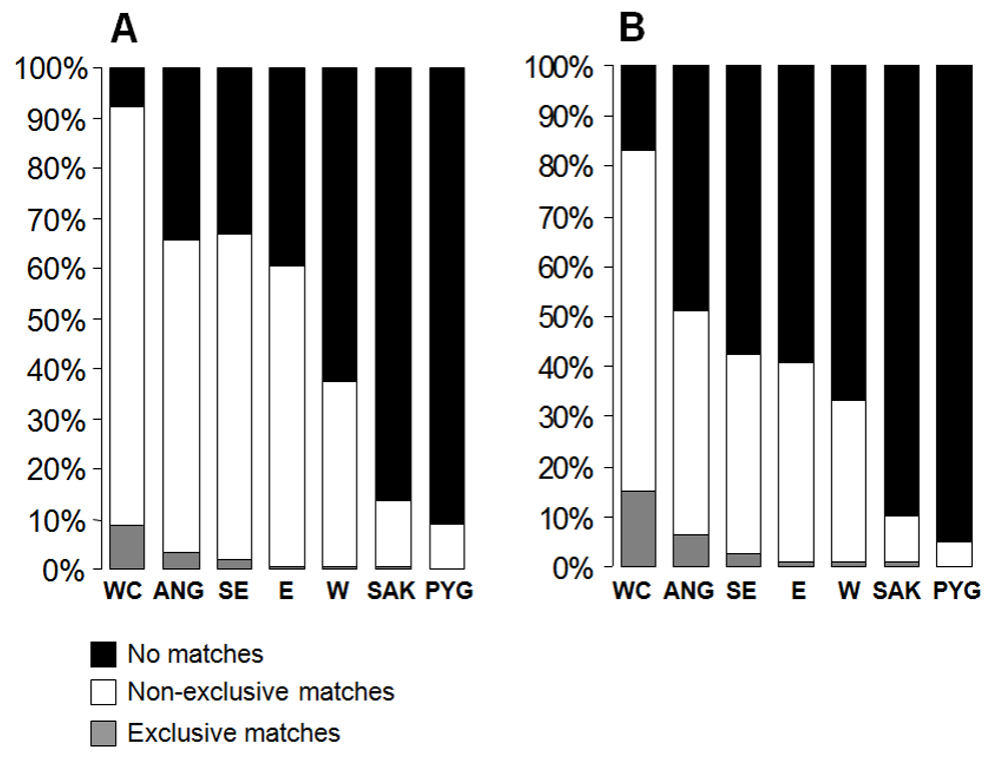
**Patterns of mtDNA lineage sharing**. Lineage sharing between individual mtDNAs (A) and haplotypes (B) from southwestern Angola and from other population groups in Africa. Only mtDNAs and haplotypes found to be shared between Namibe and at least one other African population were included in the calculations. Abbreviations are the same as Figure 2.

We evaluated the patterns of haplotype sharing using matching scores based both on individual sequences and on haplotypes. Matching scores based on individual sequences count the number of individuals from Namibe with at least one match in a given African region. Matching scores based on haplotypes, count the number of different haplotypes from Namibe with at least one match in a given African region. We found that 76% of all individual sequences and 53% of the different haplotypes sampled in Namibe match at least one sequence from elsewhere. Figure 3 shows the distribution of shared lineages across different Sub-Saharan populations. As much as 96% of all individual mtDNAs from Namibe that were found to be shared with other African populations had matches with West-Central Africa, or with regions up north in Angola (Cabinda and Luanda), which lie close to plausible migration paths between West-Central and Southwest Africa (Figure 3A). Lineage sharing with either West or East Africa is significantly lower and represents 37% and 60%, respectively, of all shared sequences from Namibe. Although lineage sharing between Namibe and the southeastern Africa is high (Figure 3), ~97% of the observed matches were found to be also shared with West-Central Africa. The link between southwestern Angola and West-Central Africa is not restricted to haplogroups that are thought to have originated in this region, like L1c or L3e [6,24]. Even shared sequences belonging to haplogroups that may trace their phylogenetic origin back to regions outside West-Central Africa were found to match sequences that occur around this area (see Additional file 7). Consideration of shared haplotypes instead of individual sequences replicates these overall sharing trends (Figure 3B).

Lineage sharing with Pygmies and southern African Khoisan-speaking peoples is low (Figure 3). Even the sequences that belong to the typical Khoe-San L0d haplogroup did not match any Khoisan-speaking population from the database. However, network analysis clearly shows that the Angolan L0d lineages are phylogenetically related to other typical Khoe-San sequences from southern Africa (see Additional file 8). We have attempted to calculate the age of the unmatched L0d lineages by estimating the average number of mutational changes to their closest southern African ancestor, using the ρ statistic (data not shown). Estimated ages were found to vary between 4816 (± 4816) and 17308 (± 9667) years.

### Admixture analysis

Although patterns of lineage sharing suggest that a substantial fraction of the mtDNA pool from southwestern Angola may have derived from West-Central Africa, important contributions from other regions cannot be firmly ruled out due to the relatively low proportion of lineages that are shared exclusively with each of several potential source areas (Figure 3). To complement the study of matching patterns, we used an explicit model of admixture [41] in which the southwestern Angolan population was considered to be a hybrid containing variable contributions from five different parental regions and populations (Table 2): West Africa, West-Central Africa, East Africa, Pygmies and the southern Africa Khoe-San (see Additional file 4). This model oversimplifies the complex demographic scenarios underlying the Bantu migrations by assuming that the Namibe pool was instantaneously created by combining different proportions of parental populations. However, the location of Namibe in a border of the Bantu expansion range, where different populations could have merged, seems to justify the use of this admixture model as an exploratory tool to assess the relative contribution of different putative source regions.

**Table 2:** Estimated admixture proportions of mtDNA lineages from southwestern Angola

	Parental Population				
Hybrid population	West-Central Africa	West Africa	East Africa	Pygmies	South Africa Khoe-San

Kuvale	0.52 (0.16)		0.16 (0.15)		0.33 (0.08)
Ganguela	0.77 (0.49)	0.05 (0.22)	0.05 (0.21)	0.10 (0.18)	0.03 (0.02)
Nyaneka-Nkhumbi	0.81 (0.09)	0.07 (0.09)			0.12 (0.09)
Ovimbundu	0.87 (0.12)	0.06 (0.11)			0.07 (0.03)

Total	0.74 (0.15)	0.04 (0.07)	0.04 (0.06)	0.05 (0.05)	0.13 (0.02)

Estimates and their standard deviations (shown between parentheses) were computed from 1000 bootstrap replications. Empty cells correspond to unsupported parental populations that were not used in admixture calculations.

Despite being associated with high standard deviations, estimates of the admixture proportions are consistent with a major (0.74 ± 0.15) contribution of West-Central Africa to the southwestern Angola mtDNA pool (Table 2). Contributions from West Africa (0.04 ± 0.07), East Africa (0.04 ± 0.06) and the Pygmies (0.05 ± 0.05) seem to have been residual and significantly lower than that from southern African Khoisan-speaking peoples (0.13 ± 0.02). Moreover, it is interesting to note that the calculated contribution from the Khoe-San in each population group shows a stepwise increase that appears to be correlated with the degree of dependence on animal husbandry of the different groups: Ganguela (0.03 ± 0.02) < Ovimbundu (0.07 ± 0.03) < Nyaneka-Nkhumbi (0.12 ± 0.03) < Kuvale (0.33 ± 0.08). The relatively low standard deviations associated with these estimates reflects the high levels of differentiation of the Khoe-San, showing that the use of an admixture model is more adequate when parental populations have remained isolated for a long time.

### Y-chromosome

#### Haplotype diversity

In accordance with the trend observed for mtDNA (Table 1), NRY diversity in STR-defined haplotypes loci was found to be lower among the Kuvale (θ_k _= 24.6) than in the Nyaneka-Humbi (θ_k _= 60.3) and the Ovimbundu (θ_k _= 61.1), revealing a consistent pattern of population size reduction and genetic drift in the Kuvale group.

### Haplogroup composition

**Figure 4:**
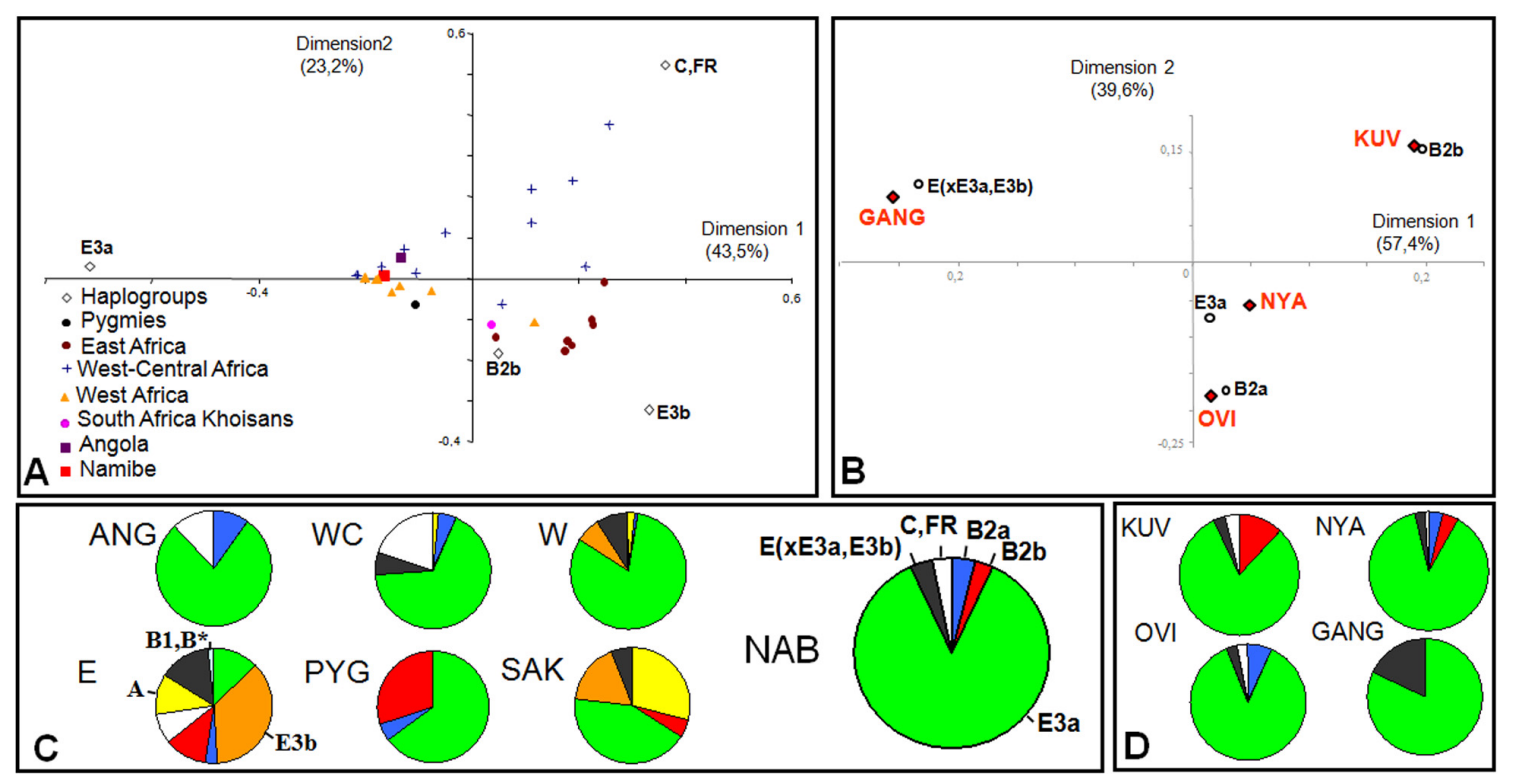
**Y-chromosome haplogroup variation in southwestern Angola and other African populations**. A) and B) Correspondence analysis plots based on haplogroup frequency profiles from several African populations (A) and different ethnolinguistic groups from Namibe (B). Percentages in parentheses indicate the total fraction of the genetic variation that was captured by each dimension. Geographic regions were defined as in Figure 1B. Angola refers to a sample from Cabinda. Namibe includes all groups sampled in this study. C) Y-chromosome haplogroup frequencies in the Namibe province and in other African population groups. D) Y-chromosome haplogroup frequencies in the four population groups sampled in the Namibe province. Abbreviations are the same as Figure 2.

Figure 4A displays the results of a correspondence analysis based on SNP-defined NRY lineages, summarizing the genetic relationships between Namibe and other sub-Saharan populations. Populations where the majority of NRY lineages belong to haplogroup E3a-M2 -including Namibe, northern Angola (Cabinda), West-Central Africa Bantu groups, West Africa and the Pygmies- are clustered together (Figures 4A and 4C). West-Central African populations located in the upper-right quadrant of the correspondence analysis plot are non-Bantu populations from Northern Cameroon with high frequencies of C, FR haplogroups. South African and East African Khoisan-speaking groups lie close to each other in the lower-right quadrant, which groups populations where B2b-M112 and E3b-M35 haplogroups are common, including all samples from East Africa (Figures 4A and 4C). A single West African pooled sample from Mali (coded as 12 in Figure 1B; see Additional file 5) is also located in the lower-right quadrant due to a high frequency of E3b-M35. However, due to paraphyly of the E3b-M35 clade [45], the sharing of E3b-M35 may not indicate a close genetic relationship between East Africa populations and the West Africa sample. When populations were classified into the same major groups defined for mtDNA (West Africa, East Africa, Southeast Africa, West-Central Africa, Pygmies and southern African Khoisan-speakers), AMOVA analysis showed that 18.2% of the variability lies between groups, 18.8% among populations within groups and 62.3% within populations (all P values <0.01). This apportionment reflects the high levels of genetic heterogeneity observed in populations from the same broad geographic area.

Although the majority (~80%) of Y-chromosome lineages in southwestern Angola belong to haplogroup E3a-M2 (Figure 4C; see Additional file 2) the distribution of the remaining lineages is not uniform across the Namibe samples (Figure 4D). The small sample from the Ganguela has E(xE3a, E3b) lineages that are less common in the other groups, while the Kuvale and the the Nyaneka-Nkhumbi carry the B2b-M112 haplogroup, which is known to be frequent both in Pygmies and Khoisan-speakers [8,9,46]. Figure 4B emphasizes the influence of the E(xE3a, E3b) and B2b minor haplogroups in separating the Ganguela and Kuvale from the Nyaneka-Nkhumbi and the Ovimbundu. This local pattern is remarkably congruent with that obtained with mtDNA (Figure 2B).

### Patterns of lineage sharing

To study the patterns of lineage sharing in the Y-chromosome, we used the populations from the comparative NRY African dataset with reported haplotype data for a common set of seven STR loci (see Additional file 5). Due to the high level of convergent evolution among NRY haplotypes based on this limited subset of STRs, the possibility of phylogenetically unrelated matches cannot be completely ruled out, as for mtDNA.

**Figure 5:**
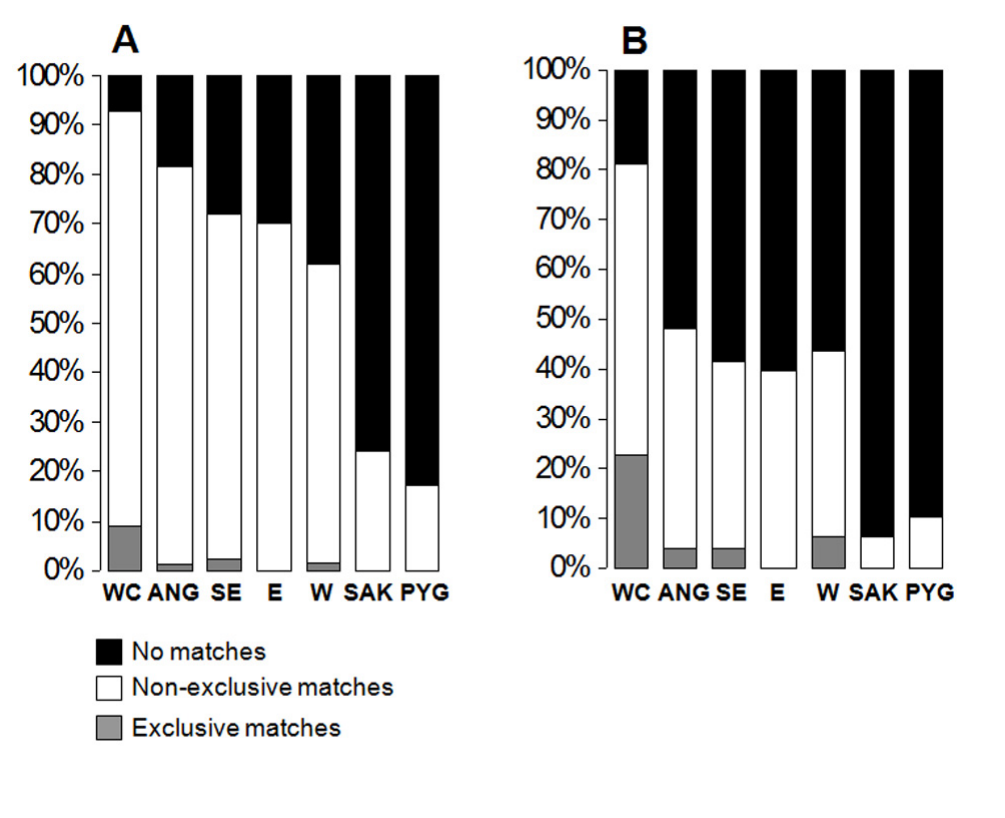
**Patterns of Y-chromosome lineage sharing**. Lineage sharing between individual Y chromosomes A) and haplotypes B) from southwestern Angola and from other population groups in Africa. Only Y chromosomes or haplotypes that were found to be shared between southwestern Angola and at least one other African population were included in the calculations. Haplotypes were defined by STR loci DYS19, DYS389I, DYS389II, DYS390, DYS391, DYS392, DYS393. Abbreviations are the same as Figure 2.

Approximately 75% of the total number of Y chromosomes and 52% of different haplotypes from southwestern Angola had at least one match with another African population. As observed for mtDNA, most Y-chromosome matches involved West-Central Africa (92% of shared Y chromosomes; 81% of shared haplotypes; Figure 5). Levels of Y-chromosome sharing with Southeast Africa were also high (72% of Y chromosomes; 42% of haplotypes), but ~97% of these individual matches (85% of different haplotypes) were shared with West-Central Africa.

Haplotypes within haplogroup B2b remained unmatched, but phylogenetic relationships inferred by network analysis suggest that these haplotypes are more likely to have been derived from Khoe-San populations than from Pygmies (see Additional file 9). Estimated ages of the unmatched B2b lineages based in the average number of mutational changes to the closest southern African ancestor were found to vary between 19445 (± 13749) and 29168 (± 20624) years.

### Admixture analysis

As for mtDNA, we used an explicit admixture model to infer the relative contributions of different African regions to the sampled southwestern Angola Y-chromosome pool. However, admixture calculations based on the frequencies of SNP-defined haplogroups lead to estimates that were associated with high standard deviations and often exceeded the 0–100% range under different combinations of parental populations. As these implausible results were likely to be due to the high similarity of haplogroup frequency profiles of West and West-Central Africa, both dominated by the E3a-M2 haplogroup, we performed a higher resolution analysis using the frequencies of haplotypes defined by a common set of 7 STR loci (see Additional file 5). At this level of resolution, the E3a-M2 haplotype subset defined by alleles 15-21-10-11-13 at loci DYS19, DYS390, DYS391, DYS392 and DYS393, which has been considered a founder lineage of Bantu expansions [7], has very different frequencies in West-Central (~0.24) and West Africa (~0.08).

To perform the STR-based admixture reanalysis, we excluded all haplotypes that did not reach a minimal 0.02 frequency threshold in at least one source region or in the whole Namibe sample. West-Central Africa and West Africa were the only supported source regions in most calculations (Table 3), with West-Central Africa providing the major admixture contribution (0.88 ± 0.19), as observed for mtDNA. Only the Nyaneka-Nkhumbi showed a signal for a possible Khoe-San contribution (0.12 ± 0.17), but we note the large standard deviation associated with this calculation.

**Table 3:** Estimated admixture proportions of Y-chromosome lineages from southwestern Angola

	Parental Population		
Hybrid population	West-Central Africa	West Africa	South Africa Khoe-San

Kuvale	0.96 (0.39)	0.04 (0.39)	
Nyaneka-Nkhumbi	0.68 (0.29)	0.20 (0.28)	0.12 (0.17)
Ovimbundu	0.99 (0.30)	0.01 (0.30)	

Total	0.88 (0.19)	0.12 (0.19)	

Estimates and their standard deviations (shown between parentheses) were computed from 1000 bootstrap replications. East Africans, and Pygmies were always unsupported as parental populations and are not shown. The Ganguela were omitted due to low sample size. Empty cells correspond to unsupported parental populations that were not used in admixture calculations.

### Lactase persistence

Given the well known association between lactose tolerance and pastoralism [47], we reasoned that the study of lactase persistence mutations in Namibe might be informative for exploring historical links between southwestern Bantu cattle herders and other pastoral communities elsewhere in Africa. To this end, we screened our sample for all SNPs that are currently known to be associated with lactase persistence in human populations. We found that the -14010C allele, which is most frequent in Nilo-Saharan and Afro-Asiatic populations from Kenya and Tanzania (32–42%, [32]), was present at lower frequencies in the Ovimbundu (1%), the Nyaneka-Nkhumbi (3%) and the Kuvale (6%). By contrast, we could not find any lactase persistence-associated allele in an additional sample of 111 individuals from several ethnolinguistic groups in Mozambique.

### Estimation of demographic parameters

Table 4 displays the estimated terms for the basic demographic parameters of the IM model using NRY and mtDNA datasets from Southwest Angola and Southeast Africa, assumed to represent the endpoints of the western and eastern branches of the Bantu migrations, respectively (see Additional file 6). Independent runs based on the NRY dataset converged on the approximate marginal posterior probability distributions for all parameters of the model (see Additional file 10). However, no convergence was found for the migration rate parameter from Southwest to Southeast Africa (m_2_, 2N_2_m_2_) under reasonable computation times using the mtDNA dataset (see Additional file 11). Therefore, we chose to present separately the outcome of individual runs with different probability density peaks for this parameter (Table 4).

**Table 4:** Estimates of demographic parameters in the Southwest and Southeast edges of the Bantu expansions

	**N1**	**N2**	**NA**	**m1**	**m2**	**2N1m1**	**2N2m2**	**t (years)**
**Y chrom**								
	10020	5510	1195	0	0	0	0	1950
	(6684–21557)	(3313–12057)	(647–2372)	(0–4 × 10^-3^)	(0–6 × 10^-3^)	(0–80.2)	(0–66.1)	(1388–2940)
**mtDNA**								
A	33212	31885	7090	2.6 × 10^-4^	5.5 × 10^-4^	17	35	25410
	(22680–44905)	(23592–46315)	(3441–16129)	(9.0 × 10^-5^-4.5 × 10^-3^)	(2.8 × 10^-4^-4.7 × 10^-3^)	(6–297)	(18–298)	(14612–39135)
B	35700	30558	7173	2.4 × 10^-4^	3.0 × 10^-3^	17	184	24978
	(24256–46066)	(23055–46315)	(3607–17041)	(5.0 × 10^-5^-3.9 × 10^-3^)	(4.5 × 10^-4^-4.8 × 10^-3^)	(4–275)	(21–296)	(13749–38319)
**Joint**								
A	6894	6150	1394	2 × 10^-3^	7 × 10^-3^	28	86	4133
	(4631–9571)	(4403–8619)	(343–2246)	(4.7 × 10^-4^-1.6 × 10^-2^)	(1.4 × 10^-3^-1.6 × 10^-2^)	(6–215)	(17–191)	(3071–13384)
B	7558	6274	1372	1 × 10^-3^	9 × 10^-3^	15	113	3981
	(5417–9929)	(4884–8578)	(933–2433)	(4.5 × 10^-4^-1.1 × 10^-2^)	(4.5 × 10^-4^-1.5 × 10^-2^)	(7–211)	(6–185)	(3147–6332)

N_1_-Current effective population size in the Southwest edge; N_2_-Current population size in the Southeast edge; N_A_-Ancestral effective population size; m1-Probability of migration from Southeast to Southwest Africa, per gene copy per generation; m_2_-Probability of migration from Southwest to Southeast Africa, per gene copy per generation; 2N_1_m_1_-Effective number of genes migrating into Southwest Africa, per generation; 2N_2_m_2_-Effective number of genes migrating into Southeast Africa, per generation; t-time since divergence from a common ancestor. 95% credibility intervals are given in parentheses. A and B show the outcome of runs with different probability density peaks for m_2_.

Estimated current population sizes (N_1 _and N_2_) are 4 to 8 fold higher than ancestral population sizes (N_A_) showing that the dispersal of Bantu speaking groups involved both significant size growth and geographic expansions. Population growth could actually have been even more marked, if bottlenecks occurring at the formation of daughter populations caused initial size reductions [48]. There are important differences in the estimates based in the mtDNA and NRY datasets. The Y-chromosome-based estimate for the current Southeast Africa population size is about one half that from the Southwest (N_1_~10000, N_2_~5000; Table 4), while current population sizes estimated from the mtDNA data are similar in both edge populations (N_1 _and N_2 _~30000; Table 4). Current population sizes based on Y-chromosome estimates lie on the lower range of reported African-specific population size, while current population size estimates from the mtDNA are in the upper range of reported values from African populations [49]. Ancestral population sizes inferred from the Y-chromosome (N_A_~1200) and mtDNA (N_A_~7100) are also different.

With regard to migration, all Y-chromosome runs consistently yielded values close to zero (see Additional file 10), corresponding to the first bin of the surveyed parameter space (Table 4). In contrast, population migration rates inferred from mtDNA are high (2N_1_m_1 _and 2N_2_m_2 _> 15; Table 4), pointing to extensive female-mediated gene flow between the west and east branches of Bantu expansions. In all cases, 2N_2_m_2 _estimates (migration from Southwest into Southeast) were found to be consistently higher than 2N_1_m_1 _(migration from Southeast into Southwest), but this observation must be regarded with caution, since 2N_2_m_2 _estimates from independent runs failed to converge to a single maximum (see Additional file 11). Although the credibility intervals obtained in different runs were quite similar, migration rate distributions were typically two-peaked. While a fraction of the runs yielded a major peak for lower 2N_2_m_2 _values (~35), in other cases the pattern reversed and the major peak corresponded to unusually high 2_2_Nm_2_values (~180) (Table 4; Additional file 11).

In order to overcome the limitations of single locus estimates, we have also generated inferences based on the combined mtDNA and NRY datasets (Table 4; Additional file 12). Joint estimates of population sizes support a 5-fold growth after population splitting (N_1_and N_2 _~7000; N_A_~1300; Table 4). Divergence time estimates were remarkably consistent with the archeological data (t = 4000 years; Table 4), while migration rates from the western to the eastern branch remained difficult to resolve, reflecting the uncertainty associated with mtDNA dataset (Table 4; Additional file 10).

## Discussion

### Southwest Angola in the African context

It is generally accepted that the mtDNA pool of Bantu speaking populations comprises a diverse set of lineages that trace their phylogeographical ancestry into three major sub-continental regions: West Africa, East Africa and West-Central Africa [6,11,24]. In spite of the growing knowledge about the ultimate regional sources of Bantu mtDNA lineages, the understanding of the major demographic processes that led to the assemblage and distribution of these diverse regional contributions among the different areas of the Bantu-speaking universe is still far from being complete. Our analysis shows that haplogroups currently associated with the Bantu mtDNA pool from southwest Angola reflect the combination of different regional contributions generally observed in most Bantu-speaking populations [6,11,24]. However, both the patterns of lineage sharing and admixture estimates from different potential source populations strongly suggest that the bulk (~75%) of mtDNA variation in southwestern Angola can be traced back just to West-Central Africa, in areas that are adjacent to the original heartland of Bantu expansions [2]. The only additional region with a significant (~13%) genetic contribution to Southwest Angola was southern Africa, indicating that most extant mtDNA variation from southwestern Angola may have simply resulted from the encounter of an offshoot of West-Central Africa with autochthonous Khoisan-speaking peoples from the south.

It is, therefore, likely that the occupation of Southwest Africa has been preceded by a period of assemblage of diverse mtDNA contributions up north, in West-Central Africa, followed by subsequent migrations from specific dispersal centers into the southwest. According to linguistic and archeological evidences, a likely dispersal center to Angola would have been located in savanna areas just south of the equatorial forest, around the Tshikapa site, where premetallurgical Bantu speakers originating in Cameroon/Gabon might have acquired iron technology and livestock from eastern Bantu peoples, before proceeding to the southwest [21,50]. The location of this center on the southern savanna edge of West-Central Africa would explain the lack of Pygmy L1c1a lineages in Angola, in contrast with the areas closer to Cameroon and Gabon where gene flow from Pygmies was more important [11].

In contrast with the collection of diverse haplogroups that is generally found in the maternal pool, the NRY haplogroup composition is highly homogeneous in most potential source areas of Bantu dispersions, due to the predominance of haplogroup E3a-M2 in West and West-Central Africa [8,9,51]. However, STR-defined haplotypes yielded sufficient resolution to allow discrimination between Y-chromosome contributions from West and West-Central Africa and reveal a link between southwestern Angola and West-Central Africa that is remarkably congruent with the results from the mtDNA dataset.

### Southwest Angola in the local and regional contexts

Despite the substantial differences between their levels of haplogroup variation, both NRY and mtDNA data concurred in showing that the populations sampled in Namibe are clustered together with other Bantu groups from elsewhere. Within the local context of southwestern Angola, the divergence of the Herero-speaking Kuvale from other population groups was found to be associated with the lack of signals of demographic expansions (Table 1), suggesting that this differentiation was shaped by increased genetic drift. Evidence for reduced levels of mtDNA diversity that are likely to have been caused by recent bottlenecks were previously described in Herero populations from Namibia and Botswana, and seem to be a pervasive feature of these groups [52,53]. However, it is difficult to know to what extent the present diversity patterns reflect the traditional semi-nomadic pastoral way of life of the Herero or were caused by population size reductions ensuing recent conflicts with colonial rulers [54-56]. Moreover, it is not clear whether genetic drift was sufficient to generate the divergence among Herero-speaking groups that is evidenced by comparisons between the Kuvale from Angola and the Herero from Namibia, which are believed to be the most representative population of the group [17,56]. In fact, while our Kuvale sample is essentially composed of mtDNA haplogroups that are commonly found in other Bantu populations from Angola (Figure 2 and Additional file 1), earlier data indicates that the Herero from Namibia display an unusually high (~50%) frequency of haplogroup L3d [57,58], which was not found in the Kuvale and is known to be much less common in most Bantu populations [6]. This pattern may imply that the broad Herero cultural division encompasses a very heterogeneous set of population groups with no obvious common origin.

A further feature of the genetic composition of the Kuvale that is not paralleled by the Herero from Namibia is their substantial levels of assimilation of Khoe-San lineages (Table 2). In fact, while Khoe-San lineages were absent in sampled Y chromosomes from the Namibian Herero [59] and may represent at most 8% of their mtDNA pool [57,58], typical Khoe-San mtDNA L0d and NRY B2b haplogroups reached 22% and 12% frequencies, respectively, in the Kuvale (Figures 2 and 4). Other sampled Bantu groups from southern Angola that are not as cattle dependent as the Kuvale exhibit much lower levels of Khoe-San lineage assimilation (Figure 2, Table 2 and Additional file 1), suggesting that most gene flow occurred between the herding Khoe peoples and the herding Bantu, probably due to the similarity of their social organization. Given the lack of shared haplotypes between Namibe L0d and B2b haplotypes and the sequences available in databases of Khoisan-speaking populations, we have estimated the ages of the introgressed lineages in order to assess the time depth underlying their present differentiation. In spite of their large uncertainty, coalescent estimates pointed to ages ranging from 4816 (± 4816) to 29168 (± 20624) years, which consistently pre-date the expected arrival time of Bantu-speaking populations to southwestern Africa [1,2]. Thus, it is likely that the divergence of these lineages occurred prior to the recent Bantu expansion. In this context, it is tempting to speculate that the unmatched Angolan L0d and B2b lineages may represent a legacy of the original speakers of Kwadi, an extinct click language remotely related with Central Khoisan that is known to have been spoken in the geographical area presently occupied by the Kuvale [60-62].

**Figure 6:**
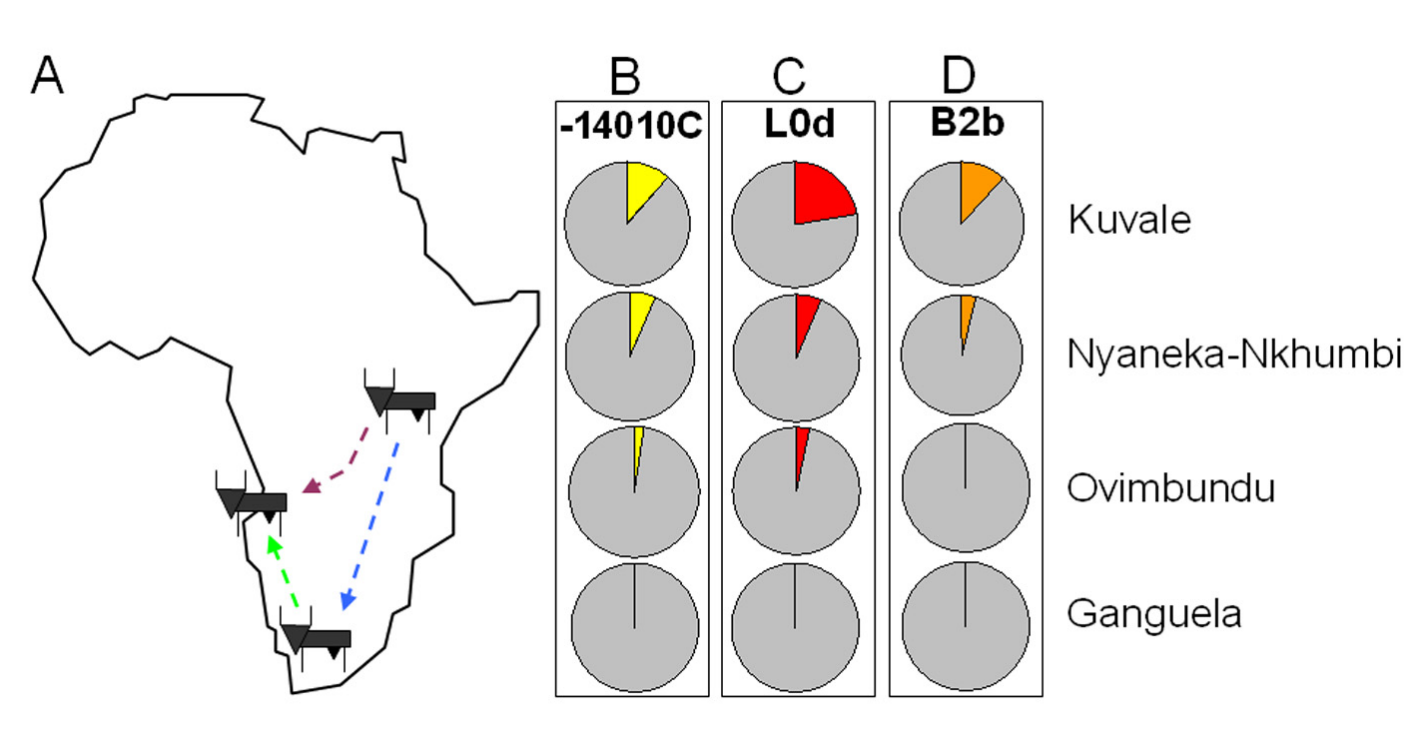
**Possible trajectories of the lactase persistence -14010C mutation from East to Southwest Africa**. A) Major hypotheses about the migration of the -14010C mutation: a direct migratory link between East and Southwest Africa (violet arrow); a Khoe mediated link, with a first contact between East African pastoralists and the herding Khoe (blue arrow) followed by subsequent transfer to Southwest Bantu pastoralists through Bantu-Khoe interactions (green arrow). B-D) carrier frequencies of the -14010C mutation (B), and typical Khoe-San mtDNA (C) and NRY lineages (D) in major ethnolinguistic groups sampled in the Namibe province.

The presence in Namibe of the -14010C lactase persistence mutation, which had only been previously found in Kenya and Tanzania [32], raises intriguing questions about the relationships between the East and Southwest African pastoral scenes. The simplest explanation for this observation would be the occurrence of a direct link between the two regions, leading to the introduction of the -14010C mutation in southwestern Angola, most likely by incoming East Bantu migrants originating in East Africa (Figure 6A). However, it is difficult to explain how the -14010C mutation could have spread from a putative Kenyan/Tanzanian center of origin into the remote areas of southwestern Angola without reaching neighboring regions in Mozambique, where no lactase persistence variants could be found. We thus favor an alternative hypothesis that takes into account the association between the frequency of the -14010C variant, the levels of Khoe-San lineage assimilation and the degree of dependence on pastoralism observed in the populations from southwestern Angola (Figure 6). According to this interpretation, the -14010C allele could have been brought to southern Africa by migrant Khoe cattle herders that had previously made contact with Nilotic or Cushitic pastoralists from East Africa. Subsequent interactions in southwestern Angola would have transferred the mutation to Bantu herders concomitantly with mtDNA and Y-chromosome lineages that are specific of the Khoe-San. Under this framework, it is conceivable that the first Bantu speakers arriving in southern Angola acquired cattle only after contact with the Khoe people. Three major lines of evidence support the Khoe-San mediated transfer of the -14010C allele. First, archeological, linguistic and ethnographic data suggest that herding Khoe peoples expanded into southern Africa 2000 years ago from areas around the upper Zambezi River, where they may have acquired pottery and livestock from East African pastoralists spreading as far south as Central Zambia [1,2,63]. Second, a recent analysis of NRY lineages from southern and eastern Africa has defined a new haplogroup (E3b1f), shared by Khoe-San and non-Bantu East Africans, whose distribution is consistent with a Bantu-unrelated demic diffusion of pastoralism from East into southern Africa, which may have started 2000 years ago [45]. Third, and as noted before [32], early reports on lactose tolerance based in physiological tests indicate that Khoisan-speaking people may have moderate levels of lactase persistence [64]. Future studies of lactase persistence based on haplotype resolution of flanking regions may shed light on the levels of genetic differentiation between variants that are presently shared by the compared populations.

### The demography of Bantu expansions

We have attempted to infer basic demographic properties of Bantu expansions using the framework of the IM model by assuming that populations located in the southwestern and southeastern edges of sub-equatorial Africa encompass the deepest branches of Bantu divergence after a common origin in West-Central Africa.

A major advantage of the IM class of models is the ability to disentangle the effects of evolutionary factors that are typically confounded in summary statistics based in equilibrium models [19,20,48]. However, like other model-based approaches, the IM framework relies on a number of simplifying assumptions that may be violated by empirical datasets. There are at least two assumptions that may influence the validity of parameter estimates in the context of the Bantu expansions. First, the model does not take into account the effects of gene flow from third party populations, whereas Bantu-speakers did undergo regional interactions with local non-Bantu groups that may distort the interpretation of inferred parameter values. Moreover, gene exchange involving unsampled demes lying between the two edge populations may affect inferences on the true patterns of migration, including the degree of asymmetrical gene flow [65]. A second limiting assumption is that the ancestral population is assumed to be unstructured and to have persisted in isolation for a long time before population splitting [66]. However the patterns of mtDNA variation suggest that the Bantu expansions might have been preceded by complex female-mediated population dynamics involving lineage assemblage in West-Central Africa and the formation of an admixed ancestral population that had no time to achieve panmixy before the expansion. It is possible that the implausibly high divergence time inferred from our mtDNA dataset (t~25000 years; Table 4) was influenced by this kind of older population structure, reflecting lack of panmixy in the ancestral population. In contrast, the more consistent divergence time estimate inferred from the Y-chromosome data (t~2000 years; Table 4) may be related to the erasure of previous ancestral variation that seems to have caused the current predominance of haplogroup E3a-M2, leading to a better fitting of the Y-chromosome data to the model. A further limitation lies in the lack of a geographical specific framework accounting for the spatial expansion of Bantu-speaking peoples.

In spite of these caveats, several consistent results could be found, showing that the analyses presented here do provide informative parameter estimates that may be contrasted in the future with other inferential frameworks and empirical datasets. Our joint estimation of the time of split between the two edges of Bantu migrations (t~4000 years; Table 4), inferred from clearly resolved posterior density peaks, is remarkably consistent with archeology-based estimates for the onset of the dispersion of Bantu speaking peoples across Africa [1,2]. On the other hand, comparisons between estimates based on the NRY and mtDNA data reveal clearly contrasting patterns between the historic demographic parameters of male and females that may account for key present-day properties of Bantu genetic variation.

Previous comparative studies on Y-chromosome and mtDNA variation in Africa provided evidence for sex biased demographic patterns, including the observation of different levels of correlation between genetic, linguistic and geographic variation [59], as well as the finding that interpopulation differentiation measured by Fst estimators is higher for the Y-chromosome than for the mtDNA in food-producing societies [67]. The latter pattern was interpreted as the result of higher migration rates and/or effective sizes in females than in males. More recently, a resequencing study of mtDNA and Y-chromosome stretches, performed in the same set of sub-Saharan African populations, has found that signals of population expansion in food-producing populations, including one combined Bantu sample, were limited to the mtDNA, while Y-chromosome data better fit models of population stationarity [68]. We found evidence for a demographic expansion both using the Y-chromosome and the mtDNA datasets (Table 4). However, since we used a different inferential framework, a different set of populations and distinct types of genetic information, it is difficult to evaluate the causes of this discrepancy. In any case, our inferences based on the IM model seem to confirm and extend the previous trends by showing that expanding Bantu females most likely had both greater population sizes (N) and higher migration rates (m) (Table 4).

As previously proposed [67,68], it is likely that cultural practices like polygyny, leading to a lower male effective size, and patrilocality, leading to a higher female migration rate, were the major driving forces underlying the observed patterns of genetic variation in current Bantu speaking populations. However, it is important to stress that differences in migration rates among Bantu populations do not necessarily imply differences in the ability to advance and settle new territory. Thus, the higher mobility of females does not mean that males advanced slower than females during the range expansion of Bantu populations, but simply that females were more likely to migrate across the different settlements that were progressively established as the Bantu dispersions unfolded.

## Conclusion

Based on patterns of lineage sharing and admixture estimates, our analysis provides evidence that most genetic variation from southwestern Angola is likely to have derived from West-Central Africa. The differences in the amount of haplogroup variation between the mtDNA and Y-chromosome data suggest that the push of Bantu peoples out of the rain forests was preceded by the assemblage of diverse mtDNA contributions in West-Central Africa, a process that was not paralleled by the Y-chromosome, in which lineage extinction must have prevailed. Estimates of demographic parameters have shown that contrasting patterns of female and male genetic variation were a pervasive feature of Bantu expansions, characterized by lower male than female effective sizes and migration rates. Local interactions between the western vanguard of the Bantu migrations and Khoisan-speaking peoples from the arid regions of the South were essentially mediated by Bantu pastoral peoples like the Herero-speaking Kuvale, who share aspects of their social organization with Khoe cattle herders from adjacent areas. We hypothesize that the East African lactase persistence -14010C mutation has been carried to southern Africa by Khoe herders who contacted East African pastoralists and subsequently transferred the mutation to Bantu cattle herders in the course of genetic interactions in the Southwest.

## Authors' contributions

JR, SB and MC conceived the study. DL carried out the molecular typing together with MC. MC and FS performed the statistical analyses. JR and SB carried out the fieldwork in southwestern Angola. MC, SB and JR have been involved in interpreting the data and drafting the manuscript. All authors revised critically the manuscript and have given final approval of the version to be published.

## Supplementary Material

Additional File 1 **MtDNA sequence data from southwestern Angola**. The table displays HVS-I and HVS-II sequence data and haplogroup classifications in population groups sampled in the Namibe province. Click here for file

Additional File 2 **NRY haplotype data from southwestern Angola**. The table displays NRY haplotypes defined by UEP and STRs in population groups sampled in the Namibe province. Click here for file

Additional File 3 **Typing procedure for lactase persistence mutations**. The file provides details on the genotyping method for the following polymorphisms associated with lactase persistence: G/C -14010; T/G -13915; C/T -13910 and C/G -13907. Click here for file

Additional File 4 **MtDNA comparative African data**. The table summarizes previously published mtDNA HVS-I datasets on African populations, here considered for comparative purposes. Click here for file

Additional File 5 **NRY comparative African data**. The table summarizes previously published NRY datasets on African populations, here considered for comparative purposes. Click here for file

Additional File 6 **The IM framework and the splitting of the western and eastern streams of Bantu migrations**. The scheme presents the basic parameters of the IM model in the context of the Bantu expansion. N_A _= population effective size of the ancestral population; N_1 _= current population size in the Southwest edge; N_2 _= current population size in the Southeast edge; m_1 _= migration rate from the eastern into the western stream; m_2 _= migration rate from the western into the eastern stream. Note that the migration parameters are identified by the destination of migrants as time goes forward. Click here for file

Additional File 7 **Patterns of mtDNA lineage sharing by haplogroup**. The figure shows the fractions of lineage sharing between southwestern Angola and other African regions for the most common mtDNA haplogroups (see Figure 3). Click here for file

Additional File 8 **Median-joining network derived from African HVS-I mtDNA sequences belonging to haplogroup L0d**. The figure shows the phylogenetic relationships between the mtDNA L0d sequences from southwestern Angola and from other African populations. Each circle represents a different haplotype. The area of the circles is proportional to the frequency of the haplotype in the populations. The branch lengths are proportional to the number of mutations separating two sequences. Click here for file

Additional File 9 **Median-joining network derived from African Y-chromosome STR-haplotypes belonging to haplogroup B2b**. The figure shows the phylogenetic relationships between Y-chromosome B2b haplotypes from southwestern Angola and from other African populations. Haplotypes were defined with a common set of 5 STR loci: DYS19, DYS389I, DYS389II, DYS390, and DYS392. The area of the circles is proportional to the frequency of the haplotype in the populations. The branch lengths are proportional to the number of mutations separating two haplotypes. Click here for file

Additional File 10 **Probability densities for the basic demographic parameters of the IM model**. The figure provides marginal posterior probability densities for independent runs of the program IMa using the Y-chromosome STR haplotype dataset (L = likelihood). Click here for file

Additional File 11 **Probability densities for the basic demographic parameters of the IM model**. The figure provides marginal posterior probability densities for independent runs of the program IMa using the mtDNA HVS-I sequence dataset (L = likelihood). Click here for file

Additional File 12 **Probability densities for the basic demographic parameters of the IM model**. The figure provides marginal posterior probability densities for independent runs of the program IMa using the joint mtDNA and Y-chromosome datasets (L = likelihood). Click here for file
