# Supplementary material for: On the edge of Bantu expansions: mtDNA, Y chromosome and lactase persistence genetic variation in southwestern Angola
Source: BMC Evol Biol. 2009 Apr 21;9:80. doi: 10.1186/1471-2148-9-80 (PMC2682489; doi:10.1186/1471-2148-9-80)
Supplement: Additional File 3 — Typing procedure for lactase persistence mutations. The file provides details on the genotyping method for the following polymorphisms associated with lactase persistence: G/C -14010; T/G -13915; C/T -13910 and C/G -13907. [file 1471-2148-9-80-S3.pdf]

Lactase persistence mutations genotyping protocol

Lactase persistence mutations G/C -14010, T/G -13915, C/T -13910 and C/G -13907 were genotyped by direct sequencing. A 359bp fragment containing all mentioned mutations and located in the intron 13 of the *MCM6* gene was amplified using primers 5'-GCAGGGCTCAAAGAACAATC-3' (forward) and 5'-TGTTGCATGTTTTTAATCTTTGG-3' (reverse). PCR reactions contained 0,5µM of each primer, 0,2mM of each deoxynucleotide triphosphate (dNTP), 750 mM Tris-HCl (pH 8.8 at 25°C), 200 mM $(NH_4)_2SO_4$, 0.1% (v/v) Tween 20, 1,5mM $MgCl_2$ and 1 U Taq polymerase. The PCR profile consisted of: 94ºC for 5 min, 35 cycles of 94ºC for 1 min, 58ºC for 1 min and 72ºC for 1min, followed by a 20-min extension at 72ºC.

Sequencing reactions were carried out using the ABI Big Dye v3.1 Ready Reaction Kit and using the protocol specified by the manufacturer (Applied Biosystems, Inc. Foster City, CA). Products were run on an ABI PRISM 3130xl sequencer and analyzed in the ABI PRISM 3130xl Genetic Analyzer software (Applied Biosystems, Inc. Foster City, CA). The resulting chromatograms were inspected for the presence/absence of the lactase mutations using MEGA4.0 software [1].

References:

1. Tamura K, Dudley J, Nei M, Kumar S: **MEGA4: Molecular Evolutionary Genetics Analysis (MEGA) software version 4.0**. *Mol Biol Evol* 2007, **24**:1596-1599.
