# Supplementary material for: On the edge of Bantu expansions: mtDNA, Y chromosome and lactase persistence genetic variation in southwestern Angola
Source: BMC Evol Biol. 2009 Apr 21;9:80. doi: 10.1186/1471-2148-9-80 (PMC2682489; doi:10.1186/1471-2148-9-80)
Supplement: Additional File 4 — MtDNA comparative African data. The table summarizes previously published mtDNA HVS-I datasets on African populations, here considered for comparative purposes. [file 1471-2148-9-80-S4.pdf]

African populations used for mtDNA comparisons.

Geographic area/Contextual samples; Pop name	Place of origin	Linguistic Affiliation	n	Code in Figure1	Reference
West Africa					
Tuareg[1,2,3]	Niger	Afro-Asiatic	23	1	[1]
Songhai[1,2,3]	Mali, Niger	Nilo-Saharan	10	2	[1]
Fulbe[1,2,3]	Benin, Burkina Faso, Níger, Nigeria	Niger Congo	60	3	[1]
Yoruba[1,2,3]	Nigeria	Niger-Congo	33	4	[1];[2]
Guinea-Bissau (various) [1,2,3]	Guinea-Bissau	Niger-Congo	372	5	[3]
Serer[1,2,3]	Senegal	Niger-Congo	23	6	[4]
Wolof[1,2,3]	Senegal	Niger-Congo	48	6	[4]
Senegalese (various) [1,2,3]	Senegal	Niger-Congo	50	6	[4]
Mandenka[1,2,3]	Senegal	Niger-Congo	110	6	[5]
Sierra Leone (various) [1,2,3]	Sierra Leone	Niger-Congo	276	7	[6]
Cabo Verde[1,2,3]	CaboVerde	Creole	292	8	[7]
West-Central Africa					
Bamileke[1,2,3]	Cameroon	Niger-Congo	48	13	[8]
Fali[1,3]	Cameroon	Niger-Congo	41	13	[9]
Fulbe[1,2,3]	Cameroon	Niger-Congo	34	3	[9]
Tali[1,3]	Cameroon	Niger-Congo	20	13	[9]
Tupuri[1,3]	Cameroon	Niger-Congo	25	13	[9]
Daba[1,3]	Cameroon	Afro-Asiatic	20	13	[9]
Mandara[1,3]	Cameroon	Afro-Asiatic	37	13	[9]
Podokwo[1,3]	Cameroon	Afro-Asiatic	39	13	[9]
Uldeme[1,3]	Cameroon	Afro-Asiatic	28	13	[9]
Bassa[1,2,3]	Cameroon	Niger-Congo (Bantu)	46	13	[9]
Bakaka[1,2,3]	Cameroon	Niger-Congo (Bantu)	50	13	[9]
Ngumba[1,2,3]	Cameroon	Niger-Congo (Bantu)	88	13	[10]
Fang[1,2,3]	Cameroon, Equatorial Guinea, Gabon	Niger-Congo (Bantu)	116	14	[10];[11]
Ewondo[1,2,3]	Cameroon	Niger-Congo (Bantu)	78	13	[8];[10]
Bubi[1,2,3]	Equatorial Guinea	Niger-Congo (Bantu)	45	16	[12]
Akele[1,2,3]	Gabon	Niger-Congo (Bantu)	48	17	[10]
Ateke[1,2,3]	Gabon	Niger-Congo (Bantu)	54	17	[10]
Benga[1,2,3]	Gabon	Niger-Congo (Bantu)	50	17	[10]
Dumba[1,2,3]	Gabon	Niger-Congo (Bantu)	47	17	[10]
Eshira[1,2,3]	Gabon	Niger-Congo (Bantu)	40	17	[10]
Eviya[1,2,3]	Gabon	Niger-Congo (Bantu)	38	17	[10]
Galoa[1,2,3]	Gabon	Niger-Congo (Bantu)	51	17	[10]
Kota[1,2,3]	Gabon	Niger-Congo (Bantu)	56	17	[10]
Makina[1,2,3]	Gabon	Niger-Congo (Bantu)	45	17	[10]
Mitsogo[1,2,3]	Gabon	Niger-Congo (Bantu)	64	17	[10]
Ndumu[1,2,3]	Gabon	Niger-Congo (Bantu)	39	17	[10]
Nzebi[1,2,3]	Gabon	Niger-Congo (Bantu)	63	17	[10]
Obamba[1,2,3]	Gabon	Niger-Congo (Bantu)	47	17	[10]
Orungu[1,2,3]	Gabon	Niger-Congo (Bantu)	20	17	[10]
Punu[1,2,3]	Gabon	Niger-Congo (Bantu)	52	17	[10]
Shake[1,2,3]	Gabon	Niger-Congo (Bantu)	51	17	[10]
Pygmies					
Babongo Pygmies[1,2,3]	Gabon	Niger-Congo	45	20	[10]

Baka Pygmies[1,2,3]	Cameroon, Gabon	Niger-Congo	127	21	[10]
Bakola Pygmies[1,2,3]	Cameroon	Niger-Congo	118	22	[10];[13]
Bakoya Pygmies[1,2,3]	Gabon	Niger-Congo	31	23	[10]
Biaka Pygmies[1,2,3]	Central African Republic	Niger-Congo	73	24	[2];[10]
Mbenzele Pygmies[1,2,3]	Central African Republic	Niger-Congo	57	25	[8]
Tikar Pygmies[1,2,3]	Cameroon	Niger-Congo	35	26	[10]
East Africa					
Etiopia[1,2,3]	Ethiopia	Afro-Asiatic	270	27	[14]
Somali[1,2,3]	Somalia	Afro-Asiatic	27	28	[1]
Burunge[1,2,3]	Tanzania	Afro-Asiatic	38	29	[13]
Turkana[1,2,3]	Kenya	Nilo-Saharan	37	30	[1]
Nairobi[2]	Kenya	Niger-Congo	100	31	[15]
Luo[1,2,3]	Kenya	Nilo-Saharan	53	32	Luiselli et al, unpublished
Nubian[1,2,3]	Nubia	Nilo-Saharan	80	33	[16]
Sudanese[1,2,3]	Sudan	Nilo-Saharan	76	34	[16]
Kikuyu[2]	Kenya	Niger-Congo (Bantu)	24	35	[1]
Turu[2]	Tanzania	Niger-Congo (Bantu)	29	36	[13]
Iraqw[1,2,3]	Tanzania	Afro-Asiatic	12	37	[17]
Datog[1,2,3]	Tanzania	Nilo-Saharan	57	38	[13]; [17]
Sukuma[2]	Tanzania	Niger-Congo (Bantu)	32	39	[13];[17]
Hadza[1,2]	Tanzania	Khoisan	145	42	[2];[13];[17]
Sandawe[1,2]	Tanzania	Khoisan	82	43	[13]
Southwest Africa					
Cabinda (Kikongo) [1,2]	Angola	Niger-Congo (Bantu)	109	44	[18]
Luanda (Kimbundu)[1,2]	Angola	Niger-Congo (Bantu)	44	45	[19]
**Namibe (various) [1,2,3,4]**	**Angola**	**Niger-Congo (Bantu)**	**365**	**46**	**Present study**
Southeast Africa					
Shona[1,2,4]	Zimbabwe	Niger-Congo (Bantu)	61	48	Luiselli et al, unpublished
Mozambique (various) [1,2]	Mozambique	Niger-Congo (Bantu)	109	47	[20]
Mozambique (various) [1,2,4]	Mozambique	Niger-Congo (Bantu)	307	47	[21]
Southern Africa Khoisans					
!Kung[1,2,3]	Botswana, South Africa	Khoisan	68	49	[2];[22]
Khwe[1,2,3]	South Africa	Khoisan	31	50	[22]
!Xun/Khwe[1,2,3]	South Africa	Khoisan	18	50	[13]

[1]Population sample used in haplogroup frequency analyses (pie charts and PC analysis);
[2]Population sample used in Lineage sharing analysis;
[3]Population sample used in admixture analyses;
[4]Population sample used in IM model and estimation of demographic parameters.

References:

1. Watson E, Bauer K, Aman R, Weiss G, von Haeseler A, Pääbo S: **mtDNA sequence diversity in Africa**. *Am J Hum Genet* 1996, **59**:437-444.
2. Vigilant L, Stoneking M, Harpending H, Hawkes K, Wilson AC: **African populations and the evolution of human mitochondrial DNA.** *Science* 1991, **253**:1503-1507.
3. Rosa A, Brehm A, Kivisild T, Metspalu E, Villems R: **MtDNA profile of West Africa Guineans: towards a better understanding of the Senegambia region.** *Ann Hum Genet* 2004, **68**: 340-352.
4. Rando JC, Pinto F, González AM, Hernández M, Larruga JM, Cabrera VM, Bandelt HJ: **Mitochondrial DNA analysis of northwest African populations reveals genetic exchanges with European, near-eastern, and sub-Saharan populations.** *Ann Hum Genet* 1998, **62**:531-550.

5. Graven L, Passarino G, Semino O, Boursot P, Santachiara-Benerecetti S, Langaney A, Excoffier L: **Evolutionary correlation between control region sequence and restriction polymorphisms in the mitochondrial genome of a large Senegalese Mandenka sample**. *Mol Biol Evol* 1995, **12**: 334-345.
6. Jackson BA, Wilson JL, Kirbah S, Sidney SS, Rosenberger J, Bassie L, Alie JA, McLean DC, Garvey WT, Ely B: **Mitochondrial DNA genetic diversity among four ethnic groups in Sierra Leone.** *Am J Phys Anthropol* 2005, **128**: 156-163.
7. Brehm A, Pereira L, Bandelt HJ, Prata MJ, Amorim A: **Mitochondrial portrait of the Cabo Verde archipelago: the Senegambian outpost of Atlantic slave trade.** *Ann Hum Genet* 2002, **66**: 49–60.
8. Destro-Bisol G, Coia V, Boschi I, Verginelli F, Cagliá A, Pascali V, Spedini G, Calafell F: **The analysis of variation of mtDNA hypervariable region 1 suggests that Eastern and Western Pygmies diverged before the Bantu expansion.** *Am Nat* 2004, **163**: 212-226.
9. Coia V, Destro-Bisol G, Verginelli F, Battaggia C, Boschi I, Cruciani F, Spedini G, Comas D, Calafell F: **Brief communication: mtDNA variation in North Cameroon: Lack of Asian lineages and implications for back migration from Asia to sub-Saharan Africa.** *Am J Phys Anthropol* 2005, **128**: 678-681.
10. Quintana-Murci L, Quach H, Harmant C, Luca F, Massonnet B, Patin E, Sica L, Mouguiama-Daouda P, Comas D, Tzur S, Balanovsky O, Kidd KK, Kidd JR, van der Veen L, Hombert JM, Gessain A, Verdu P, Froment A, Bahuchet S, Heyer E, Dausset J, Salas A, Behar DM: **Maternal traces of deep common ancestry and asymmetric gene flow between Pygmy hunter-gatherers and Bantu-speaking farmers.** *Proc Natl Acad Sci U S A* 2008, **105**: 1596-1601.
11. Pinto F, Gonzalez AM, Hernandez M, Larruga JM, Cabrera VM: **Genetic relationship between the Canary Islanders and their African and Spanish ancestors inferred from mitochondrial DNA sequences.** *Ann Hum Genet* 1996, **60**: 321-330.
12. Mateu E, Comas D, Calafell F, Pérez-Lezaun A, Abade A, Bertranpetit J: **A tale of two islands: Population history and mitochondrial DNA sequence variation of Bioko and São Tomé, Gulf of Guinea.** *Ann Hum Genet* 1997, **61**: 507-518.
13. Tishkoff SA, Gonder MK, Henn BM, Mortensen H, Knight A, Gignoux C, Fernandopulle N, Lema G, Nyambo TB, Ramakrishnan U, Reed FA, Mountain JL: **History of click-speaking populations of Africa inferred from mtDNA and Y chromosome genetic variation.** *Mol Biol Evol* 2007, **24**: 2180-2195.
14. Kivisild T, Reidla M, Metspalu E, Rosa A, Brehm A, Pennarun E, Parik J, Geberhiwot T, Usanga E, Villems R: **Ethiopian mitochondrial DNA heritage: Tracking gene flow across and around the gate of tears.** *Am J Hum Genet* 2004, **75**: 752-770.
15. Brandstätter A, Peterson CT, Irwin JA, Mpoke S, Koech DK, Parson W, Parsons TJ: **Mitochondrial DNA control region sequences from Nairobi (Kenya): inferring phylogenetic parameters for the establishment of a forensic database.** *Int J Legal Med* 2004, **118**: 294-306.
16. Krings M, Salem AE, Bauer K, Geisert H, Malek AK, Chaix L, Simon C, Welsby D, Di Rienzo A, Utermann G, Sajantila A, Pääbo S, Stoneking M: **mtDNA analysis of Nile River Valley populations: A genetic corridor or a barrier to migration?** *Am J Hum Genet* 1999, **64**: 1166-1176.
17. Knight A, Underhill PA, Mortensen HM, Zhivotovsky LA, Lin AA, Henn BM, Louis D, Ruhlen M, Mountain JL: **African Y chromosome and mtDNA divergence provides insight into the history of click languages.** *Curr Biol* 2003, **13**: 464-473.
18. Beleza S, Gusmão L, Amorim A, Carracedo A, Salas A: **The genetic legacy of western Bantu migrations.** *Hum Genet* 2005, **117**: 366-375.
19. Plaza S, Salas A, Calafell F, Corte-Real F, Bertranpetit J, Carracedo A, Comas D: **Insights into the western Bantu dispersal: mtDNA lineage analysis in Angola.** *Hum Genet* 2004 **115**: 439-447.
20. Pereira L, Macaulay V, Torroni A, Scozzari R, Prata MJ, Amorim A: **Prehistoric and historic traces in the mtDNA of Mozambique: insights into the Bantu expansions and the slave trade**. *Ann Hum Genet* 2001, **65**: 439-458.
21. Salas A, Richards M, Lareu MV, Scozzari R, Coppa A, Torroni A, Macaulay V, Carracedo A: **The making of the African mtDNA landscape.** *Am J Hum Genet* 2002, **71**: 1082-1111.
22. Chen YS, Olckers A, Schurr TG, Kogelnik AM, Huoponen K, Wallace DC: **mtDNA variation in the South African Kung and Khwe-and their genetic relationships to other African populations.** *Am J Hum Genet* 2000, **66**: 1362-1383.
