# Supplementary material for: On the edge of Bantu expansions: mtDNA, Y chromosome and lactase persistence genetic variation in southwestern Angola
Source: BMC Evol Biol. 2009 Apr 21;9:80. doi: 10.1186/1471-2148-9-80 (PMC2682489; doi:10.1186/1471-2148-9-80)
Supplement: Additional File 5 — NRY comparative African data. The table summarizes previously published NRY datasets on African populations, here considered for comparative purposes. [file 1471-2148-9-80-S5.pdf]

African populations used for Y-chromosome comparisons

Geographic area/Contextual samples; Pop name	Place of origin	Linguistic Affiliation	n	Code in Figure1	Reference
West Africa					
Tuareg[2,3]	Niger	Afro-Asiatic	9	1	[1]
Songhai[2,3]	Niger	Nilo-Saharan	5	2	[1]
Fon[1]	Benin	Niger-Congo	100	9	[2]
Rimaibe[1]	Burkina Faso	Niger Congo	37	10	[3]
Fulbe[1]	Burkina Faso	Niger-Congo	20	3	[3]
Fulbe[2,3]	Niger	Niger-Congo	6	3	[1]
Mossi[1]	Burkina Faso	Niger-Congo	49	11	[3]
Guinea Bissau (various) [2,3]	Guinea Bissau	Niger-Congo	203	5	[4],[5]
Guinea Bissau (various) [1]	Guinea Bissau	Niger-Congo	232	5	[6]
Mali (various) [1]	Mali	-[5]	44	12	[7]
Yoruba[1,2,3]	Nigeria	Niger-Congo	13	4	[8]
Senegal (various) [1]	Senegal	Niger-Congo	139	6	[9]
West-Central Africa					
Bamileke[2,3]	Cameroon	Niger-Congo	53	13	[10]
Bamileke[1]	Cameroon	Niger-Congo	133	13	[2],[3]
Bakaka[1]	Cameroon	Niger-Congo (Bantu)	12	13	[3]
Cameroon (various) [1]	Cameroon	Niger-Congo (Bantu)	14	13	[2]
Daba[1]	Cameroon	Afro-Asiatic	18	13	[3]
Ewondo[2,3]	Cameroon	Niger-Congo (Bantu)	20	13	[10]
Ewondo[1]	Cameroon	Niger-Congo (Bantu)	29	13	[3]
Tali[1]	Cameroon	Niger-Congo	15	13	[3]
Fali[1]	Cameroon	Niger-Congo	39	13	[3]
Fulbe[1]	Cameroon	Niger-Congo	17	3	[3]
Mixed Adamawa[1]	Cameroon	Niger-Congo	18	13	[3]
Mixed Chadic[1]	Cameroon	Afro-Asiatic	15	13	[3]
Mixed Nilo-Saharan[1]	Cameroon	Nilo-Saharan	9	13	[3]
Ouldeme[1]	Cameroon	Afro-Asiatic	21	13	[3]
Bangui[2,3]	Central African Republic	Niger-Congo	122	18	[11]
Lissongo[2,3]	Central African Republic	Niger-Congo (Bantu)	4	19	[1]
Bubi[2,3]	Equatorial Guinea	Niger-Congo (Bantu)	133	16	[12]
Fang[2,3]	Equatorial Guinea	Niger-Congo (Bantu)	110	14	[12]
Equatorial Guinea (various) [2,3]	Equatorial Guinea	Niger Congo	101	15	[13]
Pygmies					
Biaka Pygmies[1]	Central African Republic	Niger-Congo	20	24	[3]
Biaka Pygmies[2,3]	Central African Republic	Niger-Congo	8	24	[8]
Pygmies[2,3]	Central African Republic	Niger-Congo	20	24	[1]
East Africa					
Ethiopian Nilo-Saharan[2,3]	Ethiopia	Nilo-Saharan	40	27	[1]
Ethiopian Afro-Asiatic[2,3]	Ethiopia	Afro-Asiatic	44	27	[1]
Ethiopians[1]	Ethiopia	Afro-Asiatic	126	27	[9]

Ethiopia (various) [1]	Ethiopia	-[5]	88	27	[7]
Somalia (various) [2,3]	Somalia	-[5]	201	28	[15]
Burunge[1,2,3]	Tanzania	Afro-Asiatic	24	29	[8]
Sudan (various) [1]	Sudan	-[5]	40	34	[7]
Turu[2]	Tanzania	Niger-Congo (Bantu)	20	36	[8]
Iraqw[1]	Tanzania	Afro-Asiatic	6	37	[16]
Datog[1,2,3]	Tanzania	Nilo-Saharan	35	38	[8]
Sukuma[2]	Tanzania	Niger-Congo (Bantu)	30	39	[8]
Mbugwe[2]	Tanzania	Niger-Congo (Bantu)	14	40	[8]
Maasai[1]	Kenya	Nilo-Saharan	26	41	[14]
Hadzabe[1,2]	Tanzania	Khoisan	54	42	[8]
Hadzabe[1]	Tanzania	Khoisan	23	42	[16]
Sandawe[1,2]	Tanzania	Khoisan	67	43	[8]
Southwest Africa					
Cabinda (various) [1,2]	Angola	Niger-Congo (Bantu)	74	44	[17]
Luanda (various) [2]	Angola	Niger-Congo (Bantu)	50	45	[5]
**Namibe (various) [1,2,3,4]**	**Angola**	**Niger-Congo (Bantu)**	**236**	**46**	**Present study**
Southeast Africa					
Mozambique (various) [2,4]	Mozambique	Niger-Congo (Bantu)	112	47	[18]
South African					
Omega San[2,3]	Namibia	Khoisan	15	51	[1]
Sekele San[2,3]	South Africa	Khoisan	14	51	[1]
San[2,3]	South Africa	Khoisan	8	51	[8]
!Kung[1]	South Africa	Khoisan	64	49	[3]
Kwe[1]	South Africa	Khoisan	26	50	[3]

[1]Population sample used in haplogroup frequency analyses (pie charts and PC analysis);
[2]Population sample used in lineage sharing analysis;
[3]Population sample used in admixture analyses;
[4]Population sample used in IM model and estimation of demographic parameters.
[5]Linguistic Affiliation was not mentioned in the reference

References:

1. Pritchard JK, Seielstad M, Perez-Lezaun A, Feldman M: **Population Growth of Human Y Chromosomes: A Study of Y Chromosome Microsatellites.** *Mol Biol Evol* 1999, **16**: 1791-1798.
2. Luis JR, Rowold DJ, Regueiro M, Caeiro B, Cinnioglu C, Roseman C, Underhill PA, Cavalli-Sforza LL, Herrera RJ: **The Levant versus the Horn of Africa: evidence for bidirectional corridors of human migrations.** *Am J Hum Genet* 2004, **74**: 532-544.
3. Cruciani F, Santolamazza P, Shen P, Macaulay V, Moral P, Olckers A, Modiano D, Holmes S, Destro–Bisol G, Coia V, Wallace DC, Oefner PJ, Torroni A, Cavalli–Sforza LL, Scozzari R, Underhill PA: **A back migration from Asia to sub-Saharan Africa is supported by high-resolution analysis of human Y-chromosome haplotypes.** *Am J Hum Genet* 2002, **70**:1197–1214.
4. Rosa A, Ornelas C, Jobling MA, Brehm A, Villems R: **Y-chromosomal diversity in the population of Guinea-Bissau: a multiethnic perspective.** *BMC Evol Biol* 2007, **7**: 124.
5. Côrte-Real F, Carvalho M, Andrade L, Anjos MJ, Pestoni C, Lareu MV, Carracedo A, Vieira DN, Vide MC: **Chromosome Y STRs analysis and evolutionary aspects for Portuguese spoken countries.** In *Progress in forensic Genetics* 8; *Amsterdam.* Edited by Sensabaugh, G F P, Lincoln J, Olaisen, B: Elsevier Science; 2000: 272-274.
6. Gonçalves R, Rosa A, Freitas A, Fernandes A, Kivisild T, Villems R, Brehm A: **Y-chromosome lineages in Cabo Verde Islands witness the diverse geographic origin of its first male settlers.** *Hum Genet* 2003, **113**: 467-472.

7. Underhill PA, Shen P, Lin AA, Jin L, Passarino G, Yang WH, Kauffman E, Bonne-Tamir B, Bertranpetit J, Francalacci P, Ibrahim M, Jenkins T, Kidd JR, Mehdi SQ, Seielstad MT, Wells RS, Piazza A, Davis RW, Feldman MW, Cavalli-Sforza LL, Oefner PJ: **Y chromosome sequence variation and the history of human populations.** *Nat Genet* 2000, **26**: 358–361.
8. Tishkoff SA, Gonder MK, Henn BM, Mortensen H, Knight A, Gignoux C, Fernandopulle N, Lema G, Nyambo TB, Ramakrishnan U, Reed FA, Mountain JL: **History of click-speaking populations of Africa inferred from mtDNA and Y chromosome genetic variation.** *Mol Biol Evol* 2007, **24**: 2180-2195.
9. Semino O, Santachiara-Benerecetti AS, Falaschi F, Cavalli–Sforza LL, Underhill PA: **Ethiopians and Khoisan share the deepest clades of the human Y-chromosome phylogeny.** *Am J Hum Genet* 2002, **70**:265–268.
10. Caglià A, Tofanelli S, Coia V, Boschi I, Pescarmona M, Spedini G, Pascali V, Paoli G, Destro-Bisol G: **A study of Y-chromosome microsatellite variation in sub-Saharan Africa: a comparison between F(ST) and R(ST) genetic distances.** *Hum Biol* 2003, **75**: 313-330.
11. Lecerf M, Filali M, Grésenguet G, Ndjoyi-Mbiguino A, Le Goff J, de Mazancourt P, Bélec L: **Allele frequencies and haplotypes of eight Y-short tandem repeats in Bantu population living in Central Africa.** *Forensic Sci Int* 2007, **171**: 212-215.
12. Barrot C, Sánchez C, Xifró A, Ortega M, Mas J, Huguet E, Corbella J, Gené M: **Data for Y-chromosome haplotypes in Fang and Bubi populations from Bioko (Equatorial Guinea).** *Forensic Sci Int* 2007, **168**: e10-12.
13. Arroyo-Pardo E, Gusmão L, López-Parra AM, Baeza C, Mesa MS, Amorim A: **Genetic variability of 16 Y-chromosome STRs in a sample from Equatorial Guinea (Central Africa).** *Forensic Sci Int* 2005, **149**: 109-113.
14. Wood ET, Stover DA, Ehret C, Destro-Bisol G, Spedini G, McLeod H, Louie L, Bamshad M, Strassmann BI, Soodyall H, Hammer MF: **Contrasting patterns of Y chromosome and mtDNA variation in Africa: evidence for sex-biased demographic processes.** *Eur J Hum Genet* 2005, **13**: 867-876.
15. Hallenberg C, Simonsen B, Sanchez J, Morling N: **Y-chromosome STR haplotypes in Somalis.** *Forensic Sci Int* 2005, **151**: 317-321.
16. Knight A, Underhill PA, Mortensen HM, Zhivotovsky LA, Lin AA, Henn BM, Louis D, Ruhlen M, Mountain JL: **African Y chromosome and mtDNA divergence provides insight into the history of click languages.** *Curr Biol* 2003, **13**: 464-473.
17. Beleza S: **Phylogenetic and demographic history of two human populations revealed by the analysis of two non-recombining segments of the genome: Y-chromosome and mitochondrial DNA.** *PhD thesis*. Santiago Compostela University, 2005
18. Alves C, Gusmão L, Barbosa J, Amorim A: **Evaluating the informative power of Y-STRs: a comparative study using European and new African haplotype data.** *Forensic Sci Int* 2003, **134**: 126-133.
