# Supplementary figures and images for: On the edge of Bantu expansions: mtDNA, Y chromosome and lactase persistence genetic variation in southwestern Angola
Source: BMC Evol Biol. 2009 Apr 21;9:80. doi: 10.1186/1471-2148-9-80 (PMC2682489; doi:10.1186/1471-2148-9-80)

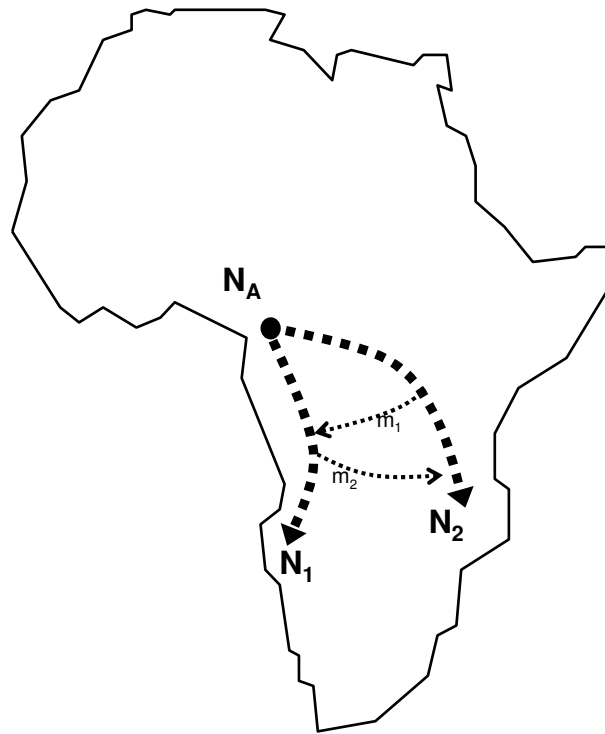

Supplement: Additional File 6 — The IM framework and the splitting of the western and eastern streams of Bantu migrations. The scheme presents the basic parameters of the IM model in the context of the Bantu expansion. NA = population effective size of the ancestral population; N1 = current population size in the Southwest edge; N2 = current population size in the Southeast edge; m1 = migration rate from the eastern into the western stream; m2 = migration rate from the western into the eastern stream. Note that the migration parameters are identified by the destination of migrants as time goes forward. [file 1471-2148-9-80-S6.pdf]

**L0a (L0a1+L0a2)**

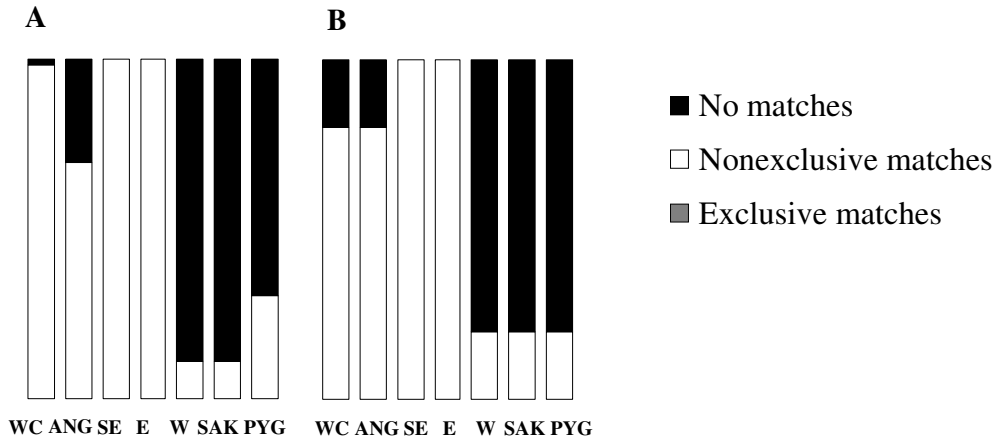

**L0a1**

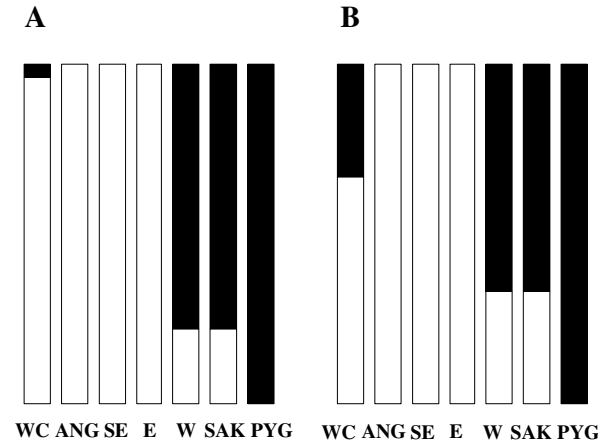

**L0a2**

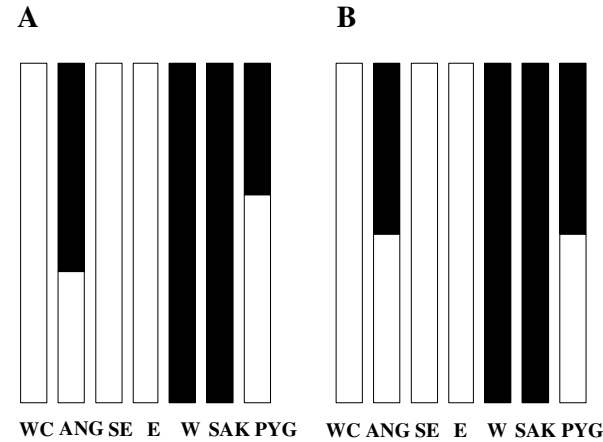

**L1c**

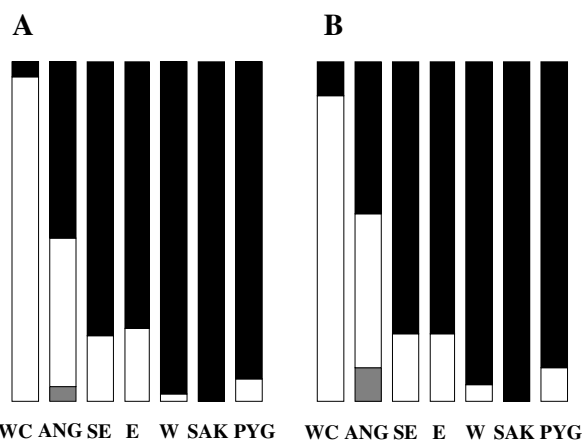

**L2a**

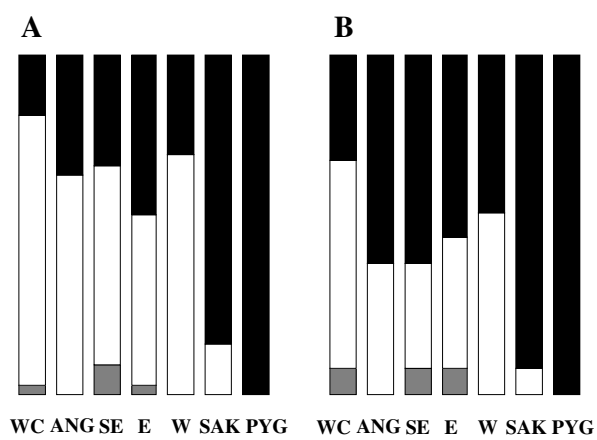

**L3e**

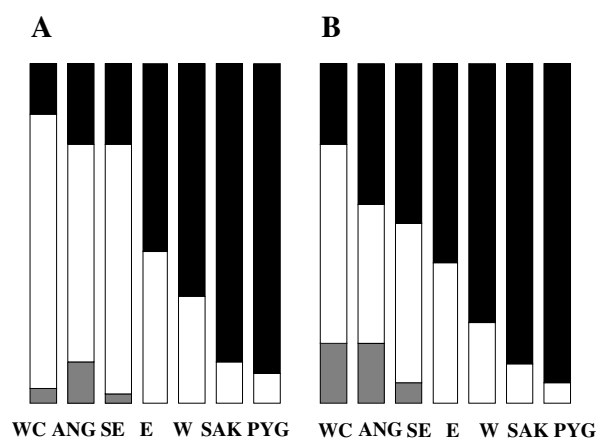

**L3f**

**A**

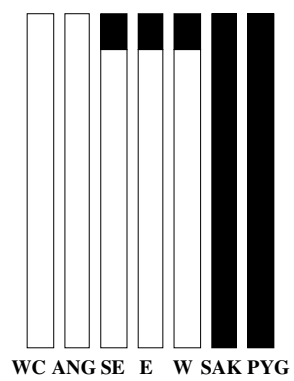

**B**

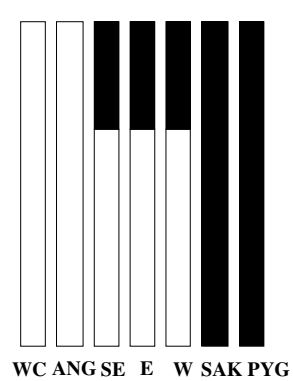

Supplement: Additional File 7 — Patterns of mtDNA lineage sharing by haplogroup. The figure shows the fractions of lineage sharing between southwestern Angola and other African regions for the most common mtDNA haplogroups (see Figure 3). [file 1471-2148-9-80-S7.pdf]

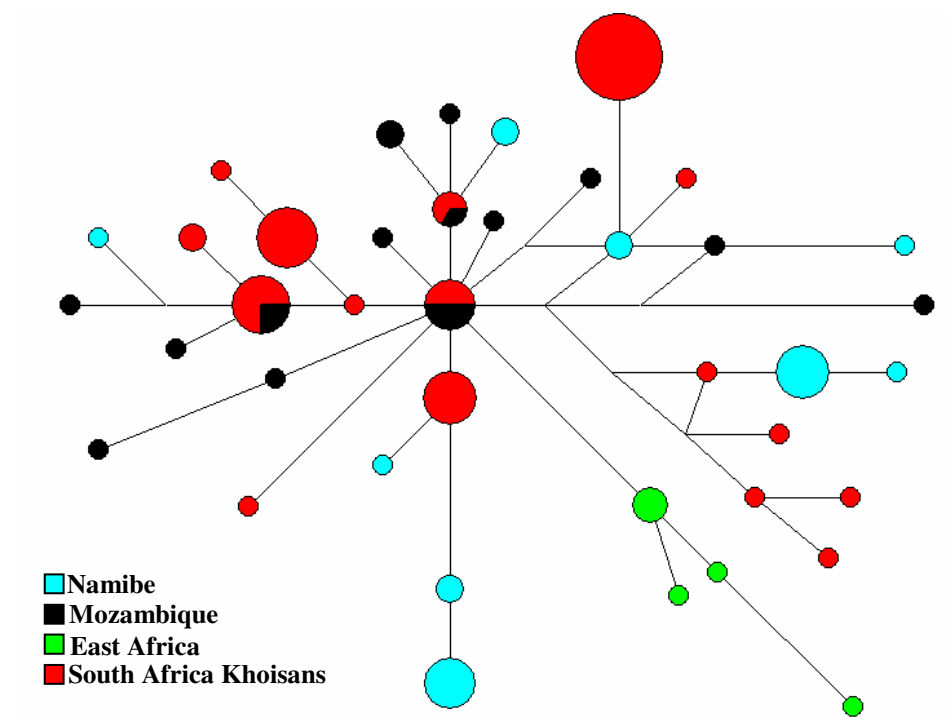

Supplement: Additional File 8 — Median-joining network derived from African HVS-I mtDNA sequences belonging to haplogroup L0d. The figure shows the phylogenetic relationships between the mtDNA L0d sequences from southwestern Angola and from other African populations. Each circle represents a different haplotype. The area of the circles is proportional to the frequency of the haplotype in the populations. The branch lengths are proportional to the number of mutations separating two sequences. [file 1471-2148-9-80-S8.pdf]

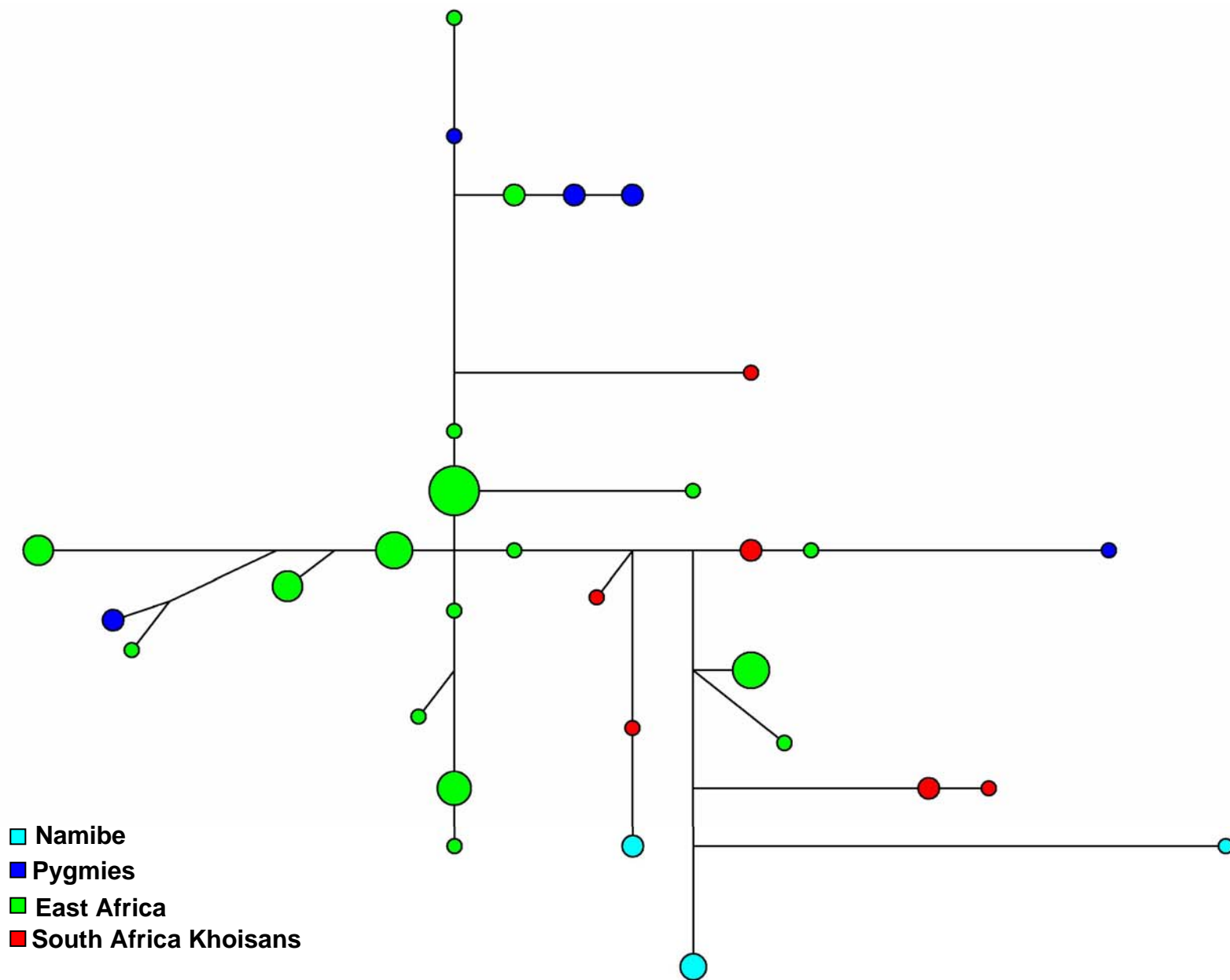

Supplement: Additional File 9 — Median-joining network derived from African Y-chromosome STR-haplotypes belonging to haplogroup B2b. The figure shows the phylogenetic relationships between Y-chromosome B2b haplotypes from southwestern Angola and from other African populations. Haplotypes were defined with a common set of 5 STR loci: DYS19, DYS389I, DYS389II, DYS390, and DYS392. The area of the circles is proportional to the frequency of the haplotype in the populations. The branch lengths are proportional to the number of mutations separating two haplotypes. [file 1471-2148-9-80-S9.pdf]

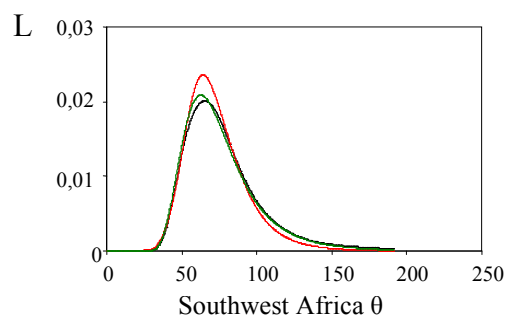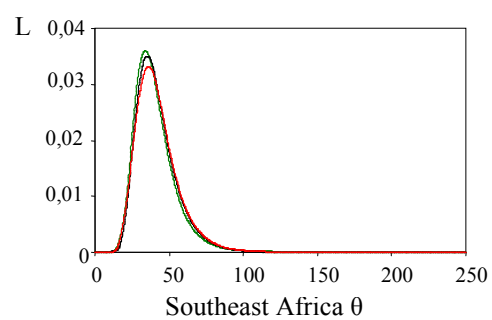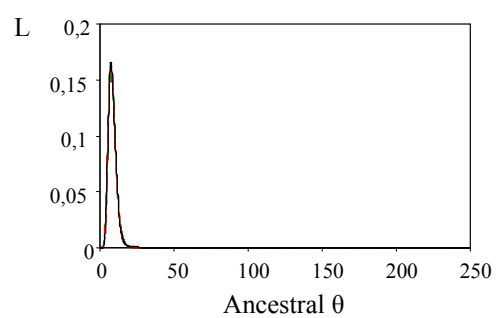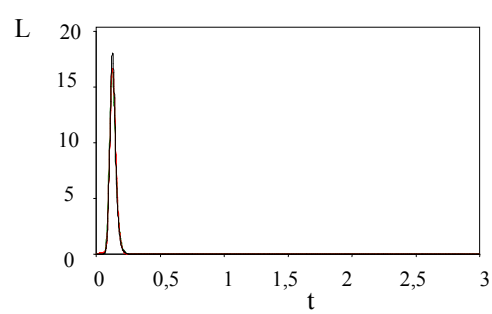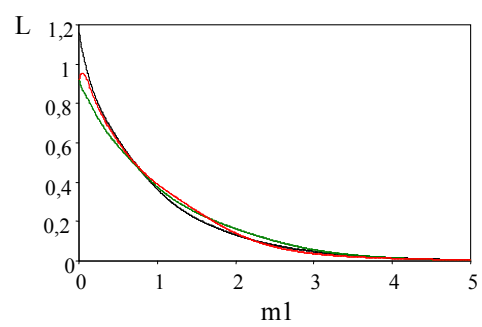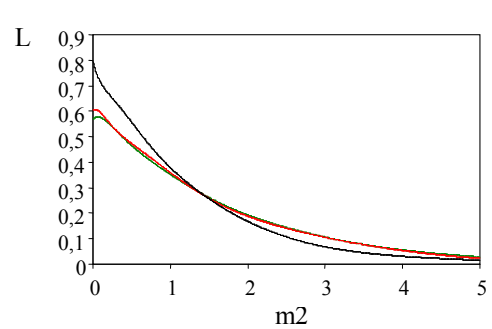

Supplement: Additional File 10 — Probability densities for the basic demographic parameters of the IM model. The figure provides marginal posterior probability densities for independent runs of the program IMa using the Y-chromosome STR haplotype dataset (L = likelihood). [file 1471-2148-9-80-S10.pdf]

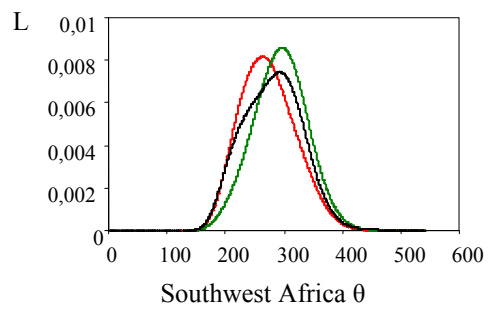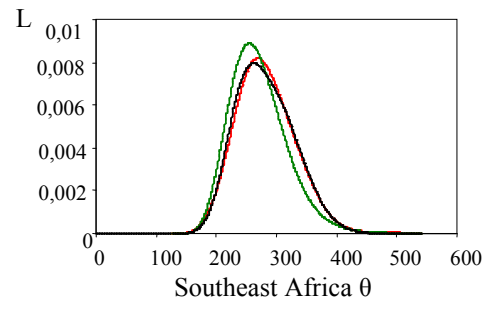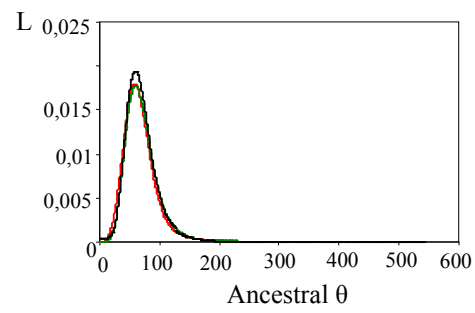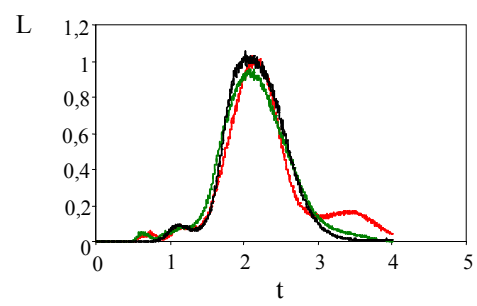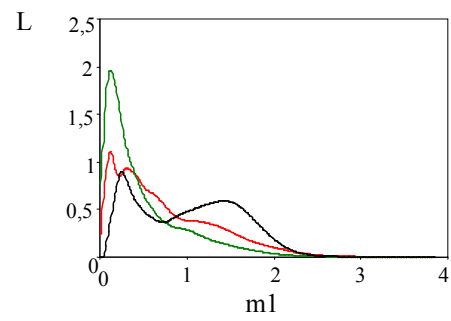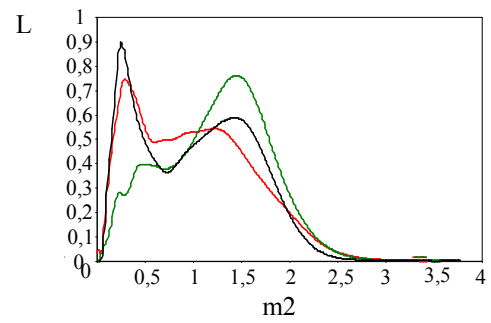

Supplement: Additional File 11 — Probability densities for the basic demographic parameters of the IM model. The figure provides marginal posterior probability densities for independent runs of the program IMa using the mtDNA HVS-I sequence dataset (L = likelihood). [file 1471-2148-9-80-S11.pdf]

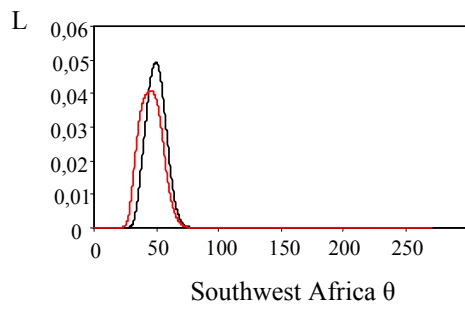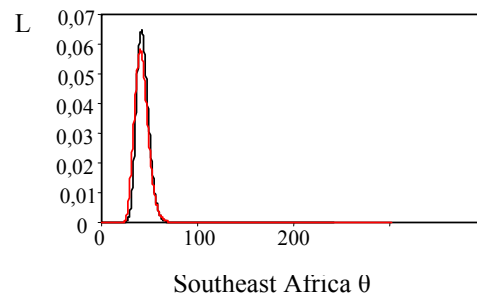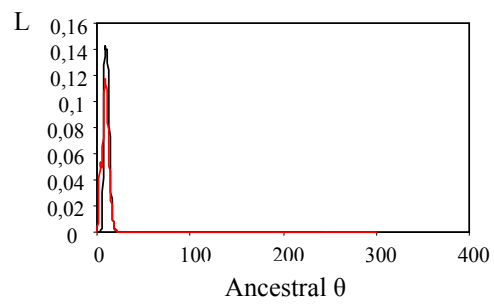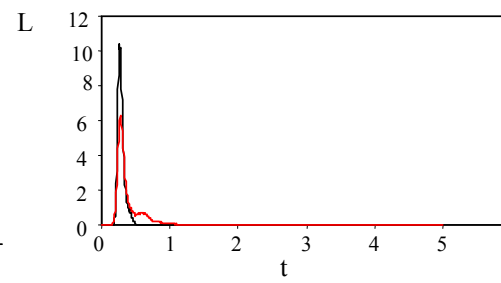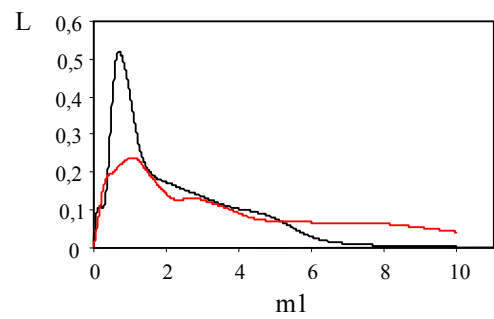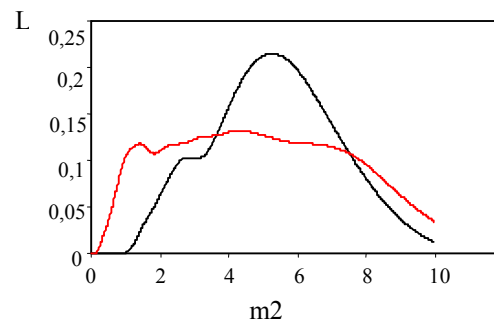

Supplement: Additional File 12 — Probability densities for the basic demographic parameters of the IM model. The figure provides marginal posterior probability densities for independent runs of the program IMa using the joint mtDNA and Y-chromosome datasets (L = likelihood). [file 1471-2148-9-80-S12.pdf]
